# High resolution chlorophyll- *a* in-situ fluorescence sensors versus in-vitro chlorophyll- *a* measurements in mesocosms with contrasting nutrient and temperature treatments

**DOI:** 10.12688/openreseurope.17146.1

**Published:** 2024-04-12

**Authors:** Eti E. Levi, Erik Jeppesen, Jens C. Nejstgaard, Thomas A. Davidson

**Affiliations:** 1 Department of Ecoscience & WATEC, Aarhus University, Aarhus, Central Denmark Region, 8000, Denmark; 2 Limnology Laboratory, Department of Biological Sciences and Centre for Ecosystem Research and implementation (EKOSAM), Middle East Technical University, Ankara, 06800, Turkey; 3 Institute of Marine Sciences, Middle East Technical University, Mersin, 33731, Turkey; 4 Sino-Danish Centre for Education and Research, University of the Chinese Academy of Sciences, Beijing, 100190, China; 5 Institute for Ecological Research and Pollution Control of Plateau Lakes, School of Ecology and Environmental Science, Yunnan University, Kunming, Yunnan, 650091, China; 6 Department of Plankton and Microbial Ecology, Leibniz-Institute of Freshwater Ecology and Inland Fisheries, Stechlin, 16775, Germany

**Keywords:** phytoplankton bloom detection, phycocyanin fluorescence, in-vivo method, spectrophotometry, high frequency data, time series, mesocosm

## Abstract

Harmful algal blooms (HABs) are a significant threat to freshwater ecosystems, and monitoring for changes in biomass is therefore important. Fluorescence in-situ sensors enable rapid and high frequency real-time data collection and have been widely used to determine chlorophyll- *a* (Chla) concentrations that are used as an indicator of the total algal biomass. However, conversion of fluorescence to equivalent Chla concentrations is often complicated due to biofouling, phytoplankton composition and the type of equipment used. Here, we validated measurements from 24 Chla and 12 phycocyanin (cyanobacteria indicator) fluorescence in-situ sensors (Cyclops-7F, Turner Designs) against spectrophotometrically (in-vitro) determined Chla and tested a data-cleaning procedure for eliminating data errors and impacts of non-photochemical quenching. The test was done across a range of freshwater plankton communities in 24 mesocosms (i.e. experimental tanks) with a 2x3 (high and low nutrient x ambient, IPCC-A2 and IPCC-A2+50% temperature scenarios) factorial design. For most mesocosms (tanks), we found accurate (r ^2^ ≥ 0.7) calibration of in-situ Chla fluorescence data using simple linear regression. An exception was tanks with high in-situ phycocyanin fluorescence, for which multiple regressions were employed, which increased the explained variance by >16%. Another exception was the low Chla concentration tanks (r ^2^ < 0.3). Our results also show that the high frequency in-situ fluorescence data recorded the timing of sudden Chla variations, while less frequent in-vitro sampling sometimes missed these or, when recorded, the duration of changes was inaccurately determined. Fluorescence in-situ sensors are particularly useful to detect and quantify sudden phytoplankton biomass variations through high frequency measurements, especially when using appropriate data-cleaning methods and accounting for factors that can impact the fluorescence readings.

## Plain language summary

Harmful algal blooms (HABs) may pose a significant threat to freshwater ecosystems and to animal and human health. Therefore, it is important to monitor changes in algal biomass. Traditional methods, while effective, lack the ability to provide rapid, high-frequency, real-time data. In-situ fluorescence sensors, specifically designed to measure chlorophyll- *a* (total phytoplankton indicator) and phycocyanin (Blue-green algae indicator), offer a promising solution. However, challenges such as biofouling, temporal changes in phytoplankton composition, and equipment variations complicate the conversion of fluorescence data into equivalent chlorophyll- *a* concentrations.

Our study aimed to validate measurements from 24 chlorophyll- *a* and 12 phycocyanin fluorescence in-situ sensors (Cyclops-7F, Turner Designs). We compared these measurements against spectrophotometrically determined (in-vitro method) chlorophyll- *a* concentrations. Additionally, we tested a data-cleaning procedure to eliminate errors caused by different sources, such as light. The validation and testing were conducted at Lemming Experimental Mesocosm site (Denmark), in 24 experimental tanks (mesocosms) representing 2 different nutrient levels and 3 temperature scenarios.

This study underlines that high-frequency in-situ fluorescence sensors can be useful, only if the user is aware of the possible interacting factors that can influence fluorescence readings (e.g. turbidity, daylight). Therefore, in-situ fluorescence sensors, when properly calibrated and validated, offer a valuable tool for monitoring harmful algal blooms. The high-frequency data provides insights into sudden variations in phytoplankton biomass, demonstrating the potential for improved real-time understanding of freshwater ecosystems.

## Introduction

Anthropogenic activities and global climate change pose a significant threat to the ecological status of freshwater ecosystems, resulting in e.g. eutrophication, which in turn alters biological community structures, including phytoplankton, and causes higher risk of harmful algal blooms (HAB) ( Jeppesen *et al.*, 2014). HABs threaten human and animal health when the waterbody is used for drinking water (e.g. Gatz, 2019). Phytoplankton is, moreover, of key importance in the nutrient cycling and energy flow and is a key metric for evaluating the water quality in lakes in the EU Water Framework Directive (WFD) ( Poikane *et al.*, 2015).

There are several ways to collect information on phytoplankton in waterbodies. Three of the most frequently used sampling/analysis methods are microscopy (e.g. Hillebrand *et al.*, 1999), pigment analysis with HPLC ( Roy *et al.*, 2011) and various forms of flowcytometry ( Lombard *et al.*, 2019). These methods are especially useful when detailed information on phytoplankton species or groups is required. Alternatively, spectrophotometric or fluorometric analyses of chlorophyll- *a* (Chla) are used as proxies for total phytoplankton biovolume. All these methods are time consuming to a varying degree. They are also prone to human error, need expertise and require transport to a laboratory for analysis. The most important disadvantage, however, is the fact that the number of samples that can be retrieved and analysed is limited, often resulting in low spatial and temporal resolution, and leaving short-term changes unnoticed ( Zamyadi *et al.*, 2012). Moreover, such ex-situ analyses do not provide the real-time information that may be needed for functional societal services such as effective HAB monitoring and warning systems.

An alternative is to use various in-situ fluorescence methods to obtain high frequency (HF) and real-time measurements of Chla and/or other pigments. The majority of the Chla in eukaryotic algae is found in the strongly fluorescent photosystem II (PSII) region, while for cyanobacteria it is mostly located in the non-fluorescent PSI region. Therefore, in-situ Chla fluorescence of cyanobacteria is low, causing underestimation of extracted (in-vitro) Chla concentrations ( Campbell *et al.*, 1998; Seppälä, 2009). The main light-harvesting pigments in the PSII region of cyanobacteria are phycobilins, such as phycocyanin or phycoerythrin ( Hodges *et al.*, 2018; Seppälä, 2009; Zamyadi *et al.*, 2012). The in-situ measurement method for phycobilins is the same as for Chla, but Chla and phycobilins differ in fluorescence characteristics. Thus, Chla absorbs and emits light in the blue (ca. 440 nm) and red (ca. 680 nm) regions of the spectrum ( Egeland *et al.*, 2011), while phycocyanin, the dominant pigment in freshwater cyanobacteria, absorbs and emits light in the red-orange region, 610–630 nm and 600 – 700 nm (maximum emission 647 nm), respectively ( Zamyadi *et al.*, 2012). Phycoerythrin pigment, on the other hand, is more common in marine species, and it has an absorption peak in the 490 – 575 nm and an emission peak in the 570 – 580 nm regions ( Seppälä, 2009), though some freshwater species, like *Planktothrix rubescens*, have a high phycoerythrin content.

The fluorescence signals are dependent on phytoplankton species composition ( Bertone *et al.*, 2019; Lawrenz & Richardson, 2011) but also influenced by cell size ( Hodges *et al.*, 2018), ambient light conditions ( Carberry *et al.*, 2019; Ralph *et al.*, 2010; Rousso *et al.*, 2021), nutrient concentrations ( Kiefer, 1973b), temperature ( Lorenzen, 1966; Watras *et al.*, 2017), turbidity ( Zamyadi *et al.*, 2016), dissolved organic matter and biofouling ( Kuha *et al.*, 2020; Xing *et al.*, 2017), all of which affect the fluorescence yield per Chla (i.e. the ratio of in-vivo Chla fluorescence to extracted Chla) ( Kiefer, 1973a; Lorenzen, 1966). Moreover, Chla and phycocyanin fluorescence might interfere with each other, and the strength of the interference depends on the concentration of these pigments ( Zamyadi *et al.*, 2012). Therefore, concentrations determined by in-vivo methods are considered semi-quantitative and caution should be taken when interpreting results ( Seppälä, 2009).

Multiple calibration studies of in-situ Chla and phycocyanin fluorometer measurements have been conducted (e.g. Gregor & Maršálek, 2004; Zamyadi *et al.*, 2012), and they have emphasised the importance of eliminating or including the interference effects from turbidity ( Zamyadi *et al.*, 2012), organic matter ( Kuha *et al.*, 2020; Xing *et al.*, 2017) and ambient light ( Carberry *et al.*, 2019; Rousso *et al.*, 2021). Moreover, several studies have investigated fluorescence sensor performances by comparing measurements from field samples with single algal species cultures ( Bastien *et al.*, 2011; Hodges *et al.*, 2018; Zamyadi *et al.*, 2012). These studies have shown that frequent validation of fluorescence readings is needed and also that the success of calibration varies – both strong ( Gregor & Maršálek, 2004) and weak ( Hodges *et al.*, 2018) correlations have been observed. By employing multivariate analysis and including DOM fluorescence readings in the Chla calibration, Kuha *et al.* (2020) found a much-improved fit (increase in r ^2^ from ca. 0.5 to 0.8) between spectrophotometric and in-situ Chla fluorescence values in 14 lakes with variable trophic state. They also pointed out that using water colour, instead of DOM, gave better calibration results.

The importance of open data sources for sharing is increasingly emphasised by the scientific community, and such sources are becoming more available. However, this represents an important challenge when using in-situ fluorescence data as there is a great variety of instruments from different manufacturers with different optical and mechanical settings. Several studies have shown that raw fluorescence sensor data can differ among brands ( Hodges *et al.*, 2018; Zamyadi *et al.*, 2016), highlighting the importance of sensor calibration to achieve harmonisation of data sets from different sources to allow comparable estimation of phytoplankton biovolume across water bodies.

The objective of this study was to find ways to efficiently clean, calibrate and thus harmonise in-situ fluorescence data collected by chlorophyll- *a* and phycocyanin sensors (Cyclops 7F, Turner Designs). We took advantage of a long running mesocosm (experimental tank) based climate change experiment facility in Denmark ( Liboriussen *et al.*, 2005, including 24 fully mixed shallow mesocosms (1 m depth) with contrasting nutrient concentrations crossed with three different temperature scenarios.

## Methods

### Study site

Chlorophyll- *a* (Chla) and phycocyanin (Phyco) fluorescence in-situ sensors were deployed in October 2018 in outdoor mesocosms (from here onwards tanks) at an experimental facility established in 2003 in Denmark (for details see Liboriussen *et al.*, 2005). The facility consists of 24 stainless steel tanks (diameter = 1.9 m, tank depth = 1.5 m, water depth = 1 m) with mixed water columns and a flow-through system (a groundwater inlet and an outlet) with a water residence time of about 2.5 months. The tanks cover six different treatments consisting of three temperature scenarios crossed with two nutrient levels, each with four replicates.

The temperature treatments consist of unheated ambient temperature (AMB), IPCC climate scenario A2 (A2), which is 2–4 °C higher than the AMB, and climate scenario A2+50% (A2+), which is 4-6 °C higher than the AMB. Half of the tanks only receive nutrients via the input of groundwater, representing low nutrient (LN) treatment ( Table 1). The other tanks, which are high nutrient treatments (HN), have been loaded with nitrogen (N) and phosphorus (P) since May 2003; Na _2_HPO _4_ and Ca(NO _3_) _2_ solutions have been added every week as sources of phosphorus and nitrogen, constituting during our study period a constant dose of 2.7 mg P m ^−2^ day ^−1^ ( Liboriussen *et al.*, 2005) and 108 mg N m ^−2^ day ^−1^. Between June 2018 and June 2019, N addition (apart from the input with groundwater) to the high nutrient (HN) tanks was suspended and subsequently N loading was resumed. 

**Table 1.** Summary data from low (LN) and high (HN) nutrient tanks for October 2018–August 2020 period.

Variables		Low nutrient (LN) tanks				High nutrient (HN) tanks			
		*mean*	*median*	*min*	*max*	*mean*	*median*	*min*	*max*
Water temp	(°C)	12.9	11.5	-0.1	31.0	13.1	11.7	-0.1	30.7
pH		8.0	7.8	6.5	10.3	8.7	8.8	5.5	12.2
Dissolved oxygen	(mg L ^-1^)	11.6	11.6	0.1	20.9	12.6	12.3	0.0	30.0
Conductivity	(µS m ^-1^)	373	380	38	525	400	392	263	920
In-vitro Chla	(µg L ^-1^)	6.6	2.7	0.3	118	165	45	0.6	2959
TP	(µg L ^-1^)	30	13	1.0	906	264	217	2.0	1219
TN	(µg L ^-1^)	422	351	1.0	2411	2085	1293	1.0	9418

The tank letters A, D, F and G indicate high nutrient (HN) treatments, while B, C, E and H are low nutrient (LN) treatments. The numbers 1, 2 and 3 indicate the different temperature treatments – as ambient (AMB), A2 and A2+, respectively.

### Sampling for chlorophyll- *a*: in-vitro determination

Water samples from three points were collected from the entire water column of each tank with a 1 m long polypropylene tube sampler (d: 10 cm), which was equipped with a stopper at the bottom. Two litre sub-sample was then transferred to dark bottles for spectrophotometric Chla analysis. Depending on the concentration, between 300 and 900 mL of water was filtered through Whatman GF/C filters (diameter: 47 mm), whereafter the filters were extracted for Chla using 10 mL 96% ethanol for 6–20 hours at room temperature. Afterwards, in-vitro Chla concentrations were determined spectrophotometrically (Shimadzu UV-1800) based on absorbance measured at 665 and 750 nm ( DS 2201, 1986). The whole process in the laboratory was conducted in a darkened laboratory.

The frequency of sample collection from the HN tanks was every second week in autumn, every month in winter and every week in spring and summer. Sampling in the LN tanks was undertaken at a slightly lower frequency, i.e. every second week for the first three months (October–December 2018) and then every month for the rest of the study period until August 2020.

### Chla and Phyco fluorescence sensors: in-situ determination

i. Sensors and their setup

Stainless steel Cyclops-7F fluorescence sensors from Turner Designs were used for in-situ determination of Chla and Phyco pigments. These in-situ sensors have three gain setting, defining their sensitivity. We used two of these gains: X1, which has a lower sensitivity with a measurement range of 0–5000 mV (equivalent to 0–500 μg Chla L ^-1^), and X10, which has a higher sensitivity with a measurement range of 0–500 mV (0–50 μg Chla L ^-1^).

PVC shade caps (length: 12.7 cm, diameter: 4.3 cm) were used on each sensor to provide a fixed distance for sample measurement, in order to reduce the effect from ambient light and to minimise the number of small animals (e.g. water snails, leeches) crawling on to the sensor tips.

The sensors were connected to a Campbell Scientific (Logan, UT, USA) CR6 datalogger through a Campbell Scientific AM16/32B multiplexer, which was used to increase the number of sensors that can be connected to the datalogger.

ii. Sensor quality checks

Sensor quality checks were performed by measuring a concentration range of Rhodamine B standard solution (linearity check-1) and water samples representing specific Chla concentrations (linearity check-2), as detailed below. Moreover, blank sample measurements using deionised water were also made (AppendixB Fig. B1).

All the measurements were conducted at the tank site with the original in-situ sensor setup (i.e. Cyclops-7F sensors connected to CR6 datalogger). Each Chla and Phyco in-situ sensor fitted with a shade cap was inserted into a glass beaker filled with relevant solution, and data were recorded in the dark.

For linearity check-1, Rhodamine B (Sigma-Aldrich R6626) stock solution was prepared according to YSI Environmental (2005) by dissolving 0.05 g Rhodamine B in distilled water. This stock solution was diluted to prepare seven different concentrations of standard solutions, representing Chla concentrations between 10 and 480 µg L ^-1^ at ca. 21.5 °C ( YSI Environmental, 2005).

Linearity check-2 was conducted for a subset of the in-situ Chla sensors. For this, deionised water served as the blank (0 µg L ^-1^). A water sample from an LN tank was used to represent 5 µg L ^-1^ (1:2 diluted) and 10 µg L ^-1^ (undiluted) Chla. Rhodamine B (Turner Designs) represented concentrations of 20 µg L ^-1^ (1:2 diluted) and 40 µg L ^-1^ (undiluted) Chla. Finally, a water sample from an HN tank was used to represent Chla concentrations of 75 µg L ^-1^ (1:2 diluted) and 150 µg L ^-1^ (undiluted).

iii. Dissolved organic carbon effect check

On July 2020, approximately 20 L water sample, representing the entire water column, were collected from all tanks using a polypropylene tube sampler (d: 10 cm, L: 1 m), with a bottom stopper. For each tank, a 60 mL subsample was filtered through GF/F grade Whatman glass microfiber filters that were combusted for 2h at 550°C. Dissolved organic carbon (DOC) was measured from the filtered water samples with a TOC-L analyser (Shimadzu Corporation, Japan) using standard method from DS/EN 1484 (1997).

Five tanks, covering low to high DOC concentrations, were chosen for further analysis. Between 400 and 600 mL of water was filtered with combusted GF/F to remove phytoplankton and to keep DOC sources. Filtered water was then divided into three subsamples and stored in dark brown bottles for at least an hour. Subsequently, Rhodamine B standards (Sigma-Aldrich R6626) were added so that the concentrations of each subsample were 62.5, 125 and 500 µg Rhod L ^-1^. These Rhodamine B concentrations were assumed to represent Chla concentrations of ca. 10 µg L ^-1^, 20 µg L ^-1^ and 80 µg L ^-1^ ( YSI Environmental, 2005).

The Chla and Phyco in-situ fluorescence of the filtered water samples and the Rhodamine B-added water samples were measured using only one Chla and one Phyco sensor, each connected to a multimeter in a laboratory. These measurements were done in dark bottles in a room kept as dark as possible at room temperature (21.2 – 21.4 °C). The data from the Rhodamine B-added samples were then compared with the values of standard Rhodamine B solutions.

### Implementation of the fluorescence in-situ sensors

Chlorophyll- *a* in-situ sensors were placed in all 24 tanks, while Phyco in-situ sensors were deployed only in the HN treatments (12 tanks). The instruments were placed so that the in-situ sensors were located in the centre of the tanks and at middle depth (50 cm below the water surface/above the bottom in these fully mixed tanks). The gain settings of the in-situ sensors were adjusted so that the sensors in the HN tanks had lower sensitivity (X1 gain) and higher X10 gain than in the LN tanks. The X10 gains were changed to X1 when necessary, i.e. ideally before running into quenching problems due to high concentrations.

As the sensors were not equipped with automatic cleaning brushes, they were instead generally cleaned every second week. As an additional control, the graphs from each sensor were examined every second or third day with LoggerNet software version 4.5 (Campbell Scientific, Logan, UT, USA) and in the case of an unexpected abrupt change, the relevant sensor was cleaned again.

### In-situ sensor data collection and cleaning

Data from the fluorescence in-situ sensors were recorded every minute during the whole period, stored as ASCII files and retrieved with LoggerNet software version 4.5 (Campbell Scientific). A data-cleaning protocol was developed and consisted of several steps (AppendixA).

Photosynthetically active radiation (PAR) was measured with S-LIA-M003 Photosynthetic Light (PAR) Smart Sensor (Onset Computers, Bourne, MA, USA) and downloaded from the Hobolink website ( www.hobolink.com, Accessed in 10.01.2022). PAR values equal to one are used to select night-time in-situ fluorescence sensor data.

The water temperature of the tanks was measured every half an hour with a PR electronics temperature sensor (see Liboriussen *et al.*, 2005 for details). Because fluorescence is temperature dependent (decreases with increasing temperature), the output from the in-situ Chla sensors was corrected according to following equation by applying 20 °C as reference temperature ( www.turnerdesigns.com/technical-support, Accessed in 07.03.2023);


%ChangeinChlain-situ=Temperature(°C)*1.4%

Fluorescence signals from the in-situ Phyco sensors were temperature corrected based on the phycocyanin-extract equation by Watras *et al.* (2017). These authors used equivalent type of sensors, Cyclops-7 instead of the 7F (faster readout version used here), from the same manufacturer, and their study was based on a 5–30 °C temperature range, which overlaps with the temperature range in our study ( Table 1). The following equation was used:


$${\text{F}}_{\text{r}}={\text{F}}_{\text{m}}/(1+\text{ρ}({\text{T}}_{\text{m}}-{\text{T}}_{\text{r}}))$$

where F: fluorescence, T: temperature, r: reference, m: measured and ρ: temperature coefficient ( Watras *et al.*, 2017).

Instead of establishing specific coefficients for each HN tank, the temperature coefficient was chosen based on the commercial phycocyanin extract ( Watras *et al.*, 2017) because each tank has its own unique cyanobacteria community, which possibly changes through the year. However, it is important to note that the optimal way of correcting the sensor signals would be by developing specific coefficients for each study environment, as recommended by Watras *et al.* (2017).

### In-situ Chla sensor calibration

Fluorescence in-situ Chla sensor readings were calibrated against in-vitro Chla measurements. Linear (i.e. in-vitro Chla ~ in-situ Chla) or multiple linear regression (i.e. in-vitro Chla ~ in-situ Chla + in-situ Phyco) analysis was used to establish equations for each in-situ Chla sensor, enabling us to predict Chla concentrations as µg L ^-1^ (from here onwards “Equivalent-Chla”) based on the in-situ fluorescence data.

The reason for using multiple regression was that Phyco is the main fluorescing pigment in cyanobacteria, and its contribution to Chla fluorescence is generally low, potentially resulting in underestimation of the spectrophotometrically measured (in-vitro) Chla in water bodies ( Campbell *et al.*, 1998; Seppälä *et al.*, 2007). Therefore, the in-situ Chla fluorescence sensors in the tanks with high cyanobacteria presence were calibrated based on multiple regression. This method was not used for all the HN tanks because a high Chla fluorescence signal can also interfere with Phyco fluorescence ( Zamyadi *et al.*, 2012), causing multicollinearity (correlation coefficient > 0.6) between the fluorescence signals of Chla and Phyco.

The Cyclops-7F (Turner Designs) in-situ sensor measurement limit is 500 µg Chla L ^-1^; therefore, sampling dates exceeding this limit (in total 19 days) were excluded from the calibrations of eight of the HN tanks.

Potential outliers were identified and removed based on combined information from linear regression diagnostic plots and robust linear regression weights with MM estimation; the information was obtained by comparing the models with and without removing possible outliers. MM estimation is a robust regression method, which is resistant to outlier influence as it has a high breakdown property, i.e. the proportion of outliers that can be handled without affecting the model is high ( Susanti *et al.*, 2014). We used this method as a check to prevent removal of legitimate data points as outliers.

Residuals were calculated as the difference between in-vitro Chla concentrations and Equivalent-Chla values. Moreover, relative error was calculated as absolute residuals divided by in-vitro Chla concentrations.

Data handling (cleaning and interpolation) and regression analysis were conducted in R version 4.2.2 ( R Core Team, 2022) with the rB3 ( Muraoka & McBride, 2018) and stats ( R Core Team, 2022) packages, respectively. All the datasets used for calibration is added as underlying data ( Levi *et al.*, 2024a) and analysis code is added as extended data ( Levi, 2024), as well as the supplementary material ( Levi *et al.*, 2024b).

## Results

### Study site between October 2018 and August 2020

During the study period, average nutrient concentrations were ca. 30 and 264 µg L ^-1^ for total phosphorus (TP) and 422 and 2085 µg L ^-1^ of total nitrogen (TN) in the LN and HN treatments, respectively ( Table 1). The average in-vitro Chla concentrations of the treatments also differed substantially, being 6.6 µg L ^-1^ in the LN and 165 µg L ^-1^ in the HN treatments. The maximum in-vitro Chla concentration in the HN tanks was as high as 2959 µg L ^-1^.

### Fluorescence in-situ sensor quality check

i. Linearity check

The linearity check-1 conducted across a range of Rhodamine B (Sigma-Aldrich R6626) concentrations showed, as expected, a good fit (AppendixB, Fig. B2).

**Figure 1.**
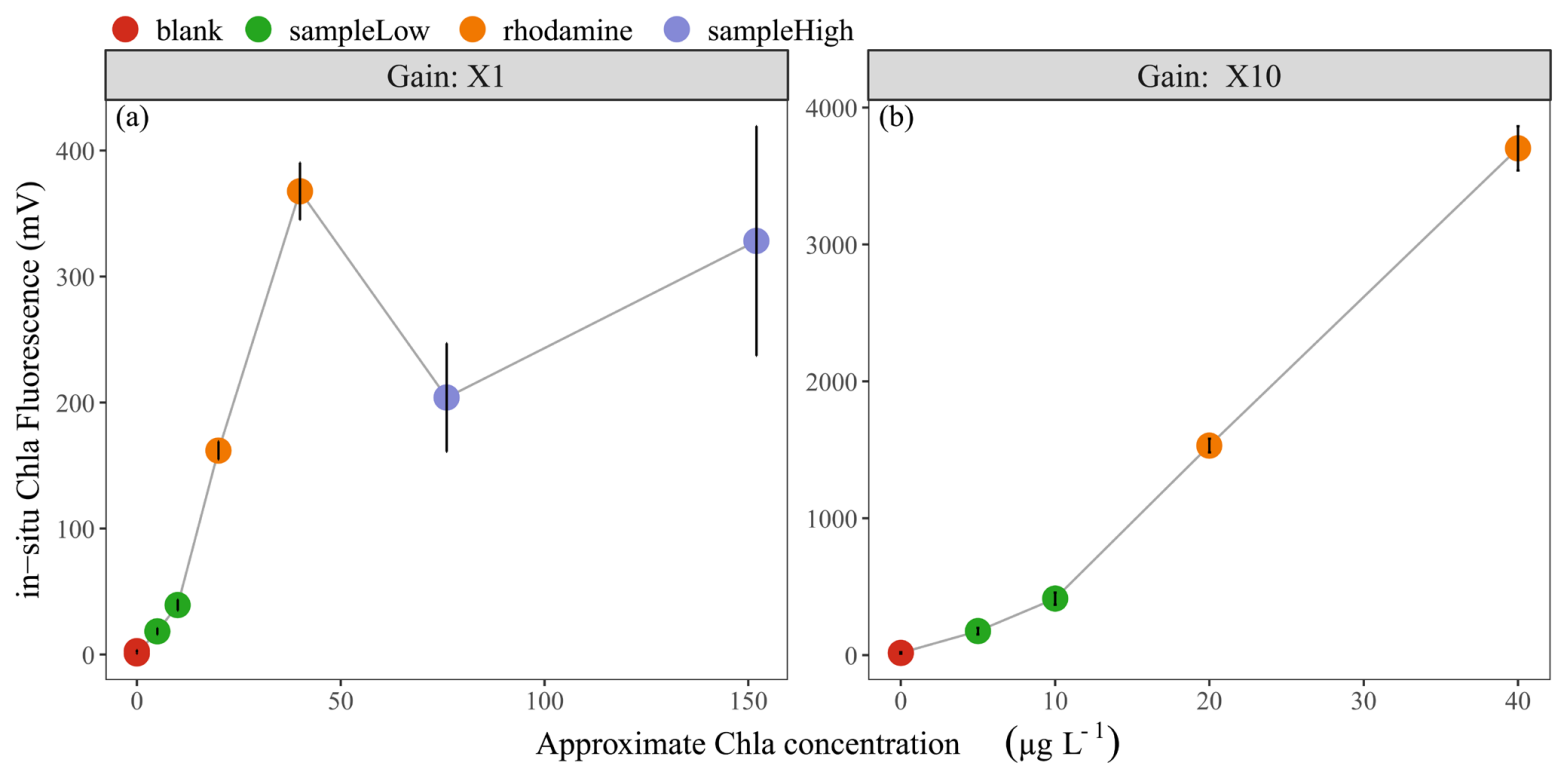
**Linearity check-2**; approximate in-vitro Chla concentrations vs fluorescence in-situ sensor (Cyclops-7F, Turner Designs) readings, showing the mean and standard deviation of ( **a**) 11 sensors with X1 gain and ( **b**) four sensors with X10 gain. In-vitro Chla concentrations for undiluted samples are; blank: 0 µg L ^-1^, sampleLow: 10 µg L ^-1^, rhodamine: 40 µg L ^-1^, sampleHigh: 150 µg L ^-1^. Note the scale differences on the axes.

The results of linearity check-2 indicated that the in-situ fluorescence measurements of Rhodamine B dye approximated our expectations with ca. 350-400 mV and 150-170 mV for non-diluted and diluted solutions, respectively ( Figure 1). The water sample with low Chla concentration (10 µg L ^-1^) showed an average of ca. 50 mV and the sample with 150 µg Chla L ^-1^ an average of 300 mV. Since the in-situ sensor range for X1 gain (0-5000 mV) is equal to 0-500 µg Chla L ^-1^, the expected values for low and high Chla samples were around 100 mV and 1500 mV, indicating that the in-situ sensor readings were approximately 2 to 5 times lower than expected ( Figure 1). Moreover, the in-situ Phyco fluorescence signal from the low concentration sample was ca. 1 mV, while relatively high values of ca. 35 mV were found for the high concentration sample.

ii. Dissolved organic carbon effect check

The impact of organic matter on Chla fluorescence readings is well documented (e.g. Kuha *et al.*, 2020). In our enclosures, however, Zhou *et al.* (2019) showed that the concentration of organic matter was relatively low and mainly derived from algae. They also demonstrated that in all the treatments, and especially in the LN tanks, a terrestrial humic-like component (C1, Ex: ≤ 230 and 340 nm/Em: 460 nm) had a lower fluorescence intensity (R.U.) compared to other fluorophores. Therefore, we did not expect to see substantial interference from DOC. 

**Figure 2.**
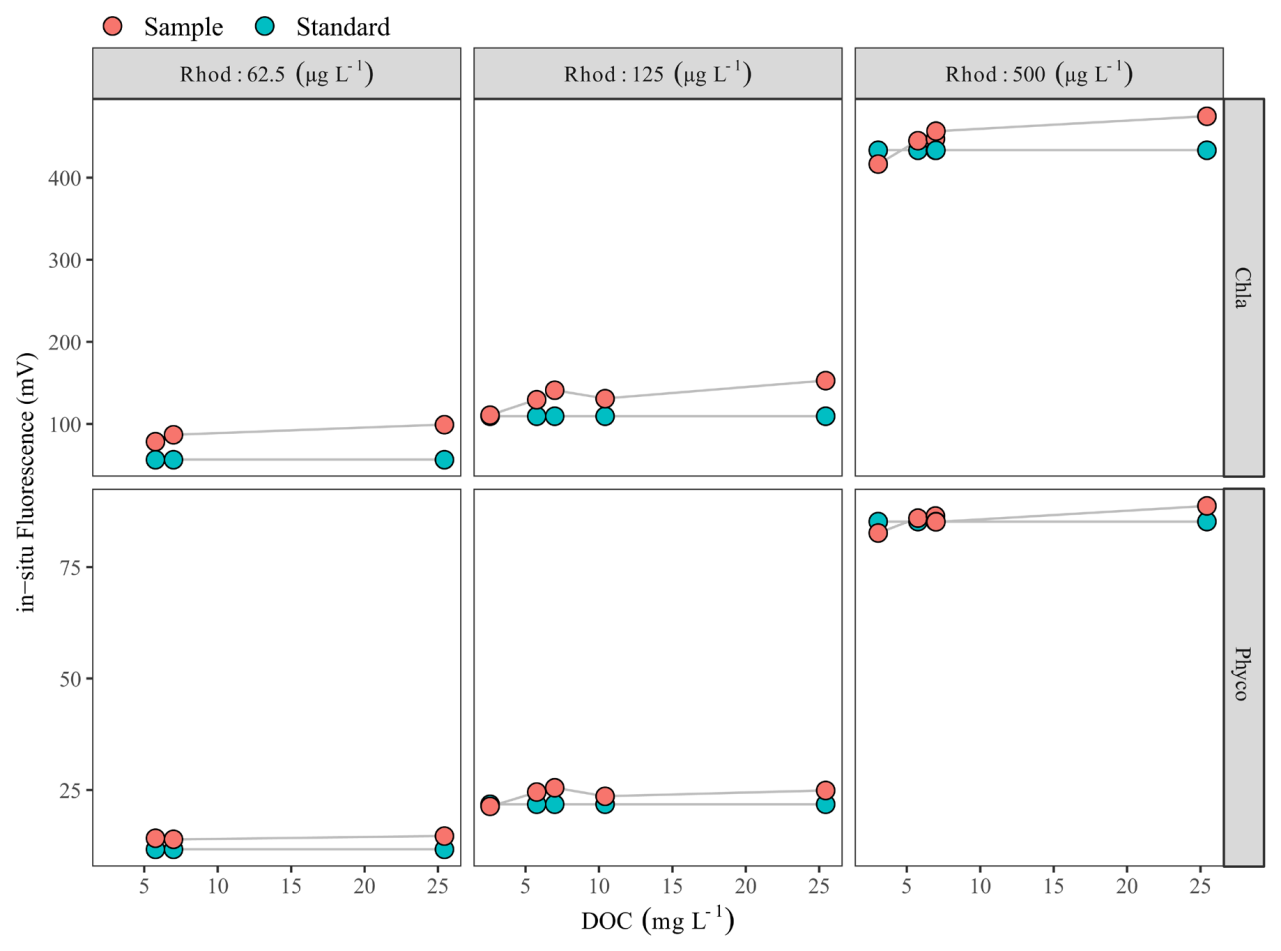
Dissolved organic carbon (DOC) effect check. DOC vs Rhodamine B-added filtered water samples (red circles) Rhodamine B standards (green circles) measured with Cyclops-7F sensors (Turner Designs). Each red circle represents a different water sample with a different DOC concentration. Note the scale difference on the y-axis.

Dissolved organic carbon (DOC) in the samples collected on 27 July 2020 ranged between 2.6 and 25.5 mg L ^-1^, with a mean of 3.7 mg L ^-1^ and 8.8 mg L ^-1^ for the LN and HN treatments, respectively ( Figure 2). Comparison of in-situ fluorescence readings between Rhodamine B standard solutions and Rhodamine B added water samples showed that the in-situ Chla fluorescence increased with increasing DOC, and at the highest DOC the difference was ca. 40 mV. However, when DOC was <5 mg L ^-1^, the impact was relatively low, with a ca. 3 mV increase at 2.6 mg DOC L ^-1^ ( Figure 2). In our case, in-situ Phyco fluorescence readings were not affected by DOC ( Figure 2). 

We collected DOC samples from all the tanks an additional four times between the end of August and the start of September 2020, and the concentration ranged between ca. 2-5 mg L ^-1^ and 4-9 mg L ^-1^ in the LN and HN tanks, respectively. These values correspond well with those recorded in summer 2016 by Zhou *et al.* (2019) (ca. 2-5 mg and 5-8 mg L ^-1^ for the LN and HN tanks, respectively), indicating rather stable DOC concentrations with occasional exceptions in the HN tanks. Therefore, we did not consider the DOC effect further.

### In-situ fluorescence data cleaning and problems

Throughout the study period, some inconsistencies were observed in the fluorescence in-situ sensor data, some of which reflected biofouling on the shade caps and electricity cuts, resulting in an unexpectedly high and gradually increasing variance (e.g. PhycoG3, Nov/2018, AppendixD page 6) and negative or missing values, respectively. Biofouling on the optics of the sensors further caused a gradual decrease in in-situ fluorescence values, following a sudden increase after sensor cleaning (e.g. PhycoG1, Date: 14-09-2019, AppendixD page 2). Filamentous algae cover around the shade caps of the in-situ sensors (e.g. ChlB3, Aug/2019, AppendixD page 9) also led to erroneously quenched data. In addition, we identified problems with the sensor pins or with the multiplexer causing structural data changes (jumps or drops) (ChlC1 and ChlE2, AppendixD pages 7 and 8). Inconsistent sudden and steady jumps or drops were also found, for which no reasons could be provided. Moreover, pond snails, snail eggs, leeches and damselfly larvae were observed on the shade caps, possibly occasionally creating questionable signal values.

Fluorescence in-situ sensor data collection over a year at high resolution (every minute, 533101 data points for each sensor) allowed data cleaning in several steps (see AppendixA). Even though the cleaning procedure worked well, it did not allow us to remove all faulty readings, such as the ones due to structural changes caused by electronic problems or the gradual decrease caused by biofouling. With this method, on average only 15 days of data out of ca. 500–670 days with measurements had to be removed (see AppendixA, for details).

### Fluorescence in-situ Chla sensor calibration for each tank

The night-time medians of fluorescence in-situ readings from each sensor were calibrated against in-vitro Chla measurements from the same tanks and days, resulting in ca. 22 – 30 calibration points for the HN tanks and ca. 18 – 25 points for the LN tanks.

**Figure 3.**
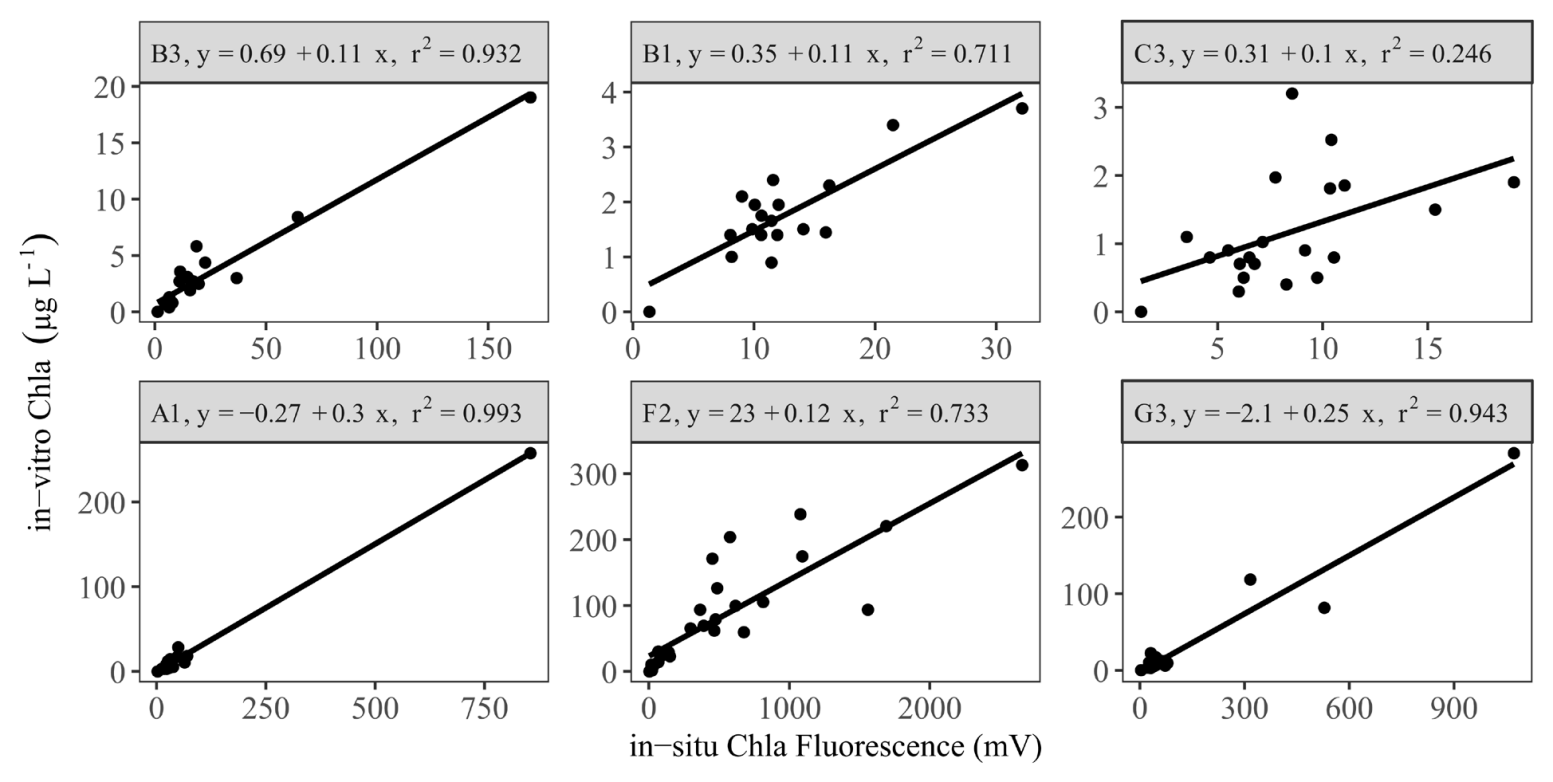
Linear regression results for a selection of low (upper panel) and high (lower panel) nutrient tanks, showing calibration equations and the coefficient of determination (r ^2^) for each tank (see AppendixB, Fig. B3 and B4 for all the tanks).

Following removal of outliers, there were generally good linear relationships (r ^2^ ≥ 0.7) between the in-vitro and in-situ measurements in the LN tanks ( Figure 3 upper panel, AppendixB, Fig. B3). An exception was the sensor from tank C3, which had a low coefficient of determination (r ^2^ ca. 0.3). This tank had relatively low in-vitro Chla concentrations throughout the study period (<10 µg L ^-1^). Similarly, two other tanks (B1 and C1) with relatively low in-vitro Chla concentrations also showed low explained variance prior to the removal of outliers (data not shown).

**Figure 4.**
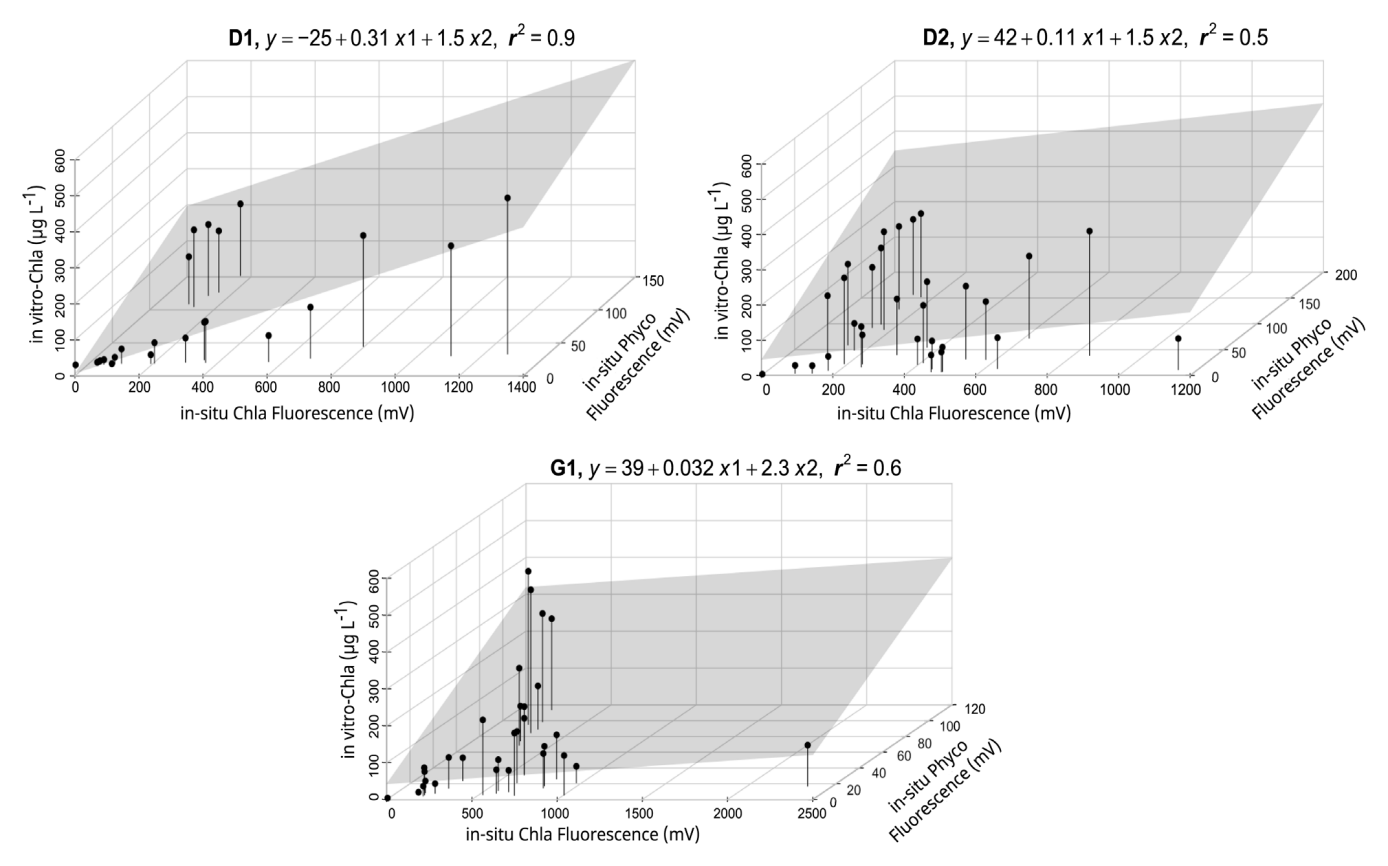
Multiple linear regression results for a selection of high nutrient (HN) tanks, showing calibration equations and the coefficient of determination (r ^2^) for each tank. The area highlighted in grey indicates the regression plane (see AppendixB, Fig. B5 for all the tanks).

The sensors in the HN tanks ( Figure 3 lower panel, AppendixB Fig. B4) generally had a high r ^2^ (> 0.6). However, high cyanobacteria concentrations in some tanks led to weaker relationships (AppendixB Fig. B5), but inclusion of Phyco night-time median readings in multiple regressions resulted in a better model fit and a 16–57% increase in explained variability (AppendixB, Fig. B5 vs B6), resulting in r ^2^ > 0.5 ( Figure 4, AppendixB Fig. B6). The calibration plots of the D1 and G1 sensors showed a high in-vitro Chla concentration either as a result of high in-situ Phyco fluorescence or high in-situ Chla fluorescence ( Figure 4).

### Comparing Equivalent-Chla and in-vitro Chla

**Figure 5.**
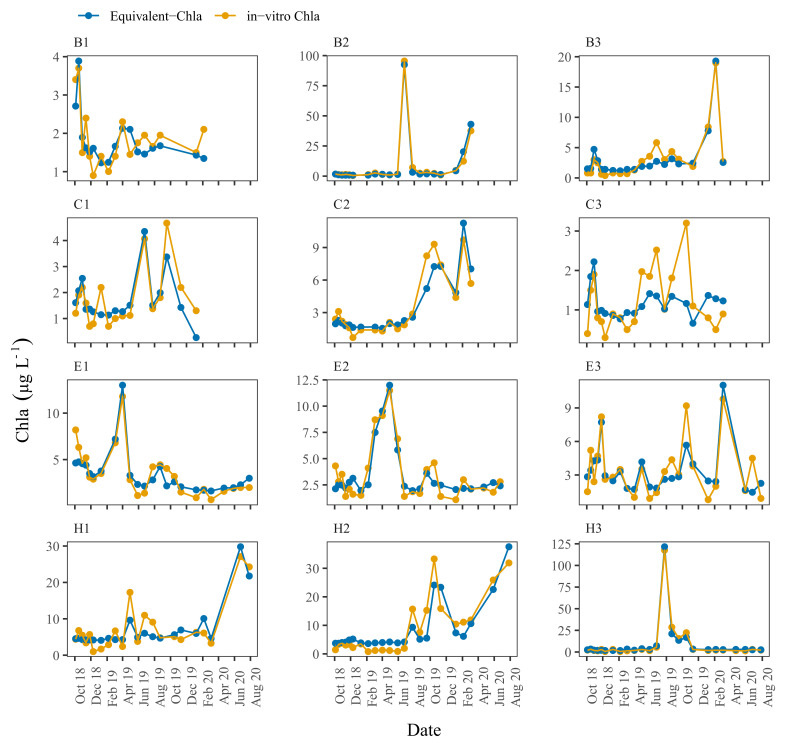
Biweekly/monthly time series plots for low nutrient (LN) tanks between October 2018 and August 2020. Note the scale differences on the y-axis.

Time series plots comparing Equivalent-Chla and in-vitro Chla showed good agreement for the LN tanks, especially for B2 and H1, where the Chla concentration range was wide (0–125 µg L ^-1^) ( Figure 5). However, some mismatch occurred, especially in the C3 and, to some extent, in B1 and C1 where in-vitro Chla concentrations were low (0.5 – 5 µg L ^-1^), being in the lowest 1-10% of the sensor range at X10 (0–50 µg L ^-1^).

**Figure 6.**
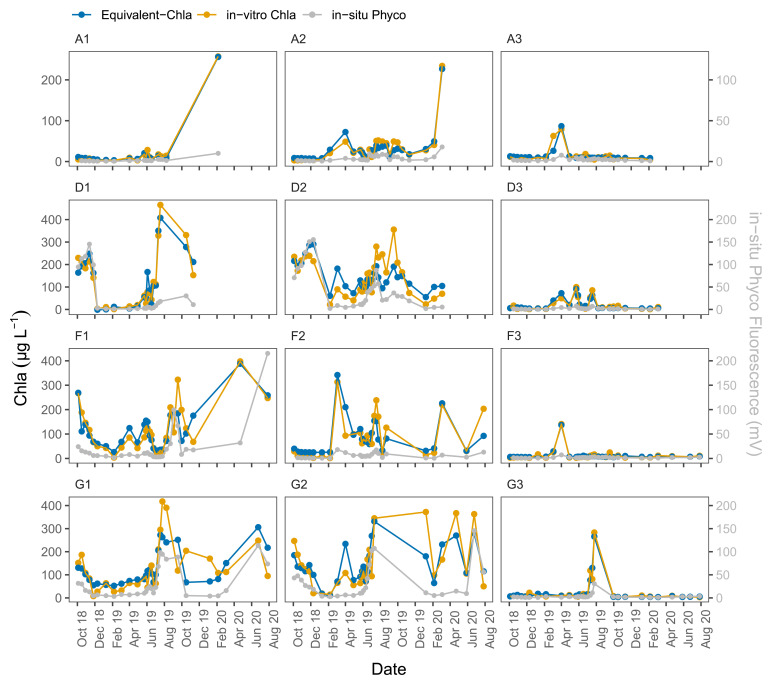
Biweekly/monthly time series plots for high nutrient (HN) tanks between October 2018 and August 2020. Note the scale and unit differences on the primary and secondary y-axis.

The time series plots of the HN tanks showed good correspondence as well ( Figure 6). In general, the sensors captured the spring/summer/autumn peaks and the decreases in the colder months. There were, some inconsistencies, however, not least in the D2 tank. At the start of the sampling period, the agreement was better for D2 where the predicted values followed the trend of in-vitro Chla, but after February-2019 the change in in-vitro Chla was not captured well by the sensors. A similar pattern was observed for G1, especially after mid-August 2019 when there was a recurring biofouling problem on the optical part of the sensor; this was obvious from the gradual decrease in in-situ fluorescence followed by a sudden increase after sensor cleaning (e.g. ChlG1 and PhycoG1, Date: 14-09-2019, AppendixD page 2). Moreover, in both of these cases in-situ Phyco fluorescence showed a higher explained variance with in-vitro Chla, with r ^2^ = 0.42 for D2 and, r ^2^ = 0.56 for G1 (data not shown) compared to in-situ Chla fluorescence.

### Accuracy of the Equivalent-Chla concentration

**Figure 7.**
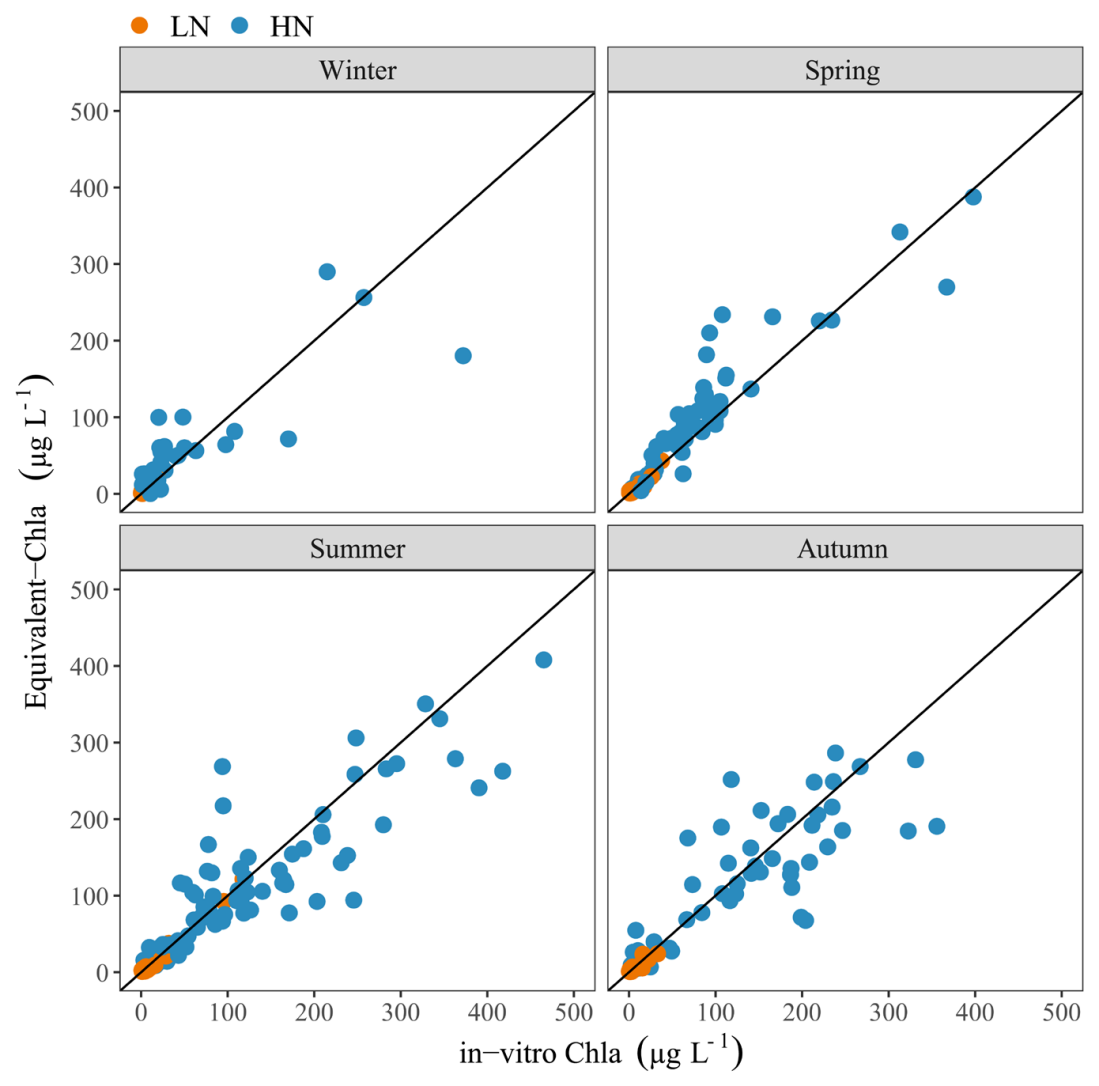
Comparison of Equivalent-Chla and in-vitro Chla concentrations, grouped according to season. Solid line indicates the 1:1 relation. Nutrient treatments are low (LN) and high (HN).

The comparison of Equivalent-Chla and in-vitro Chla showed a relatively good 1:1 relation ( Figure 7), although some deviation was observed. Especially in spring, there was a tendency to overestimation of Chla by Equivalent-Chla for both the HN and LN treatments and underestimation in the summer, particularly for the HN treatments ( Figure 7). 

**Figure 8.**
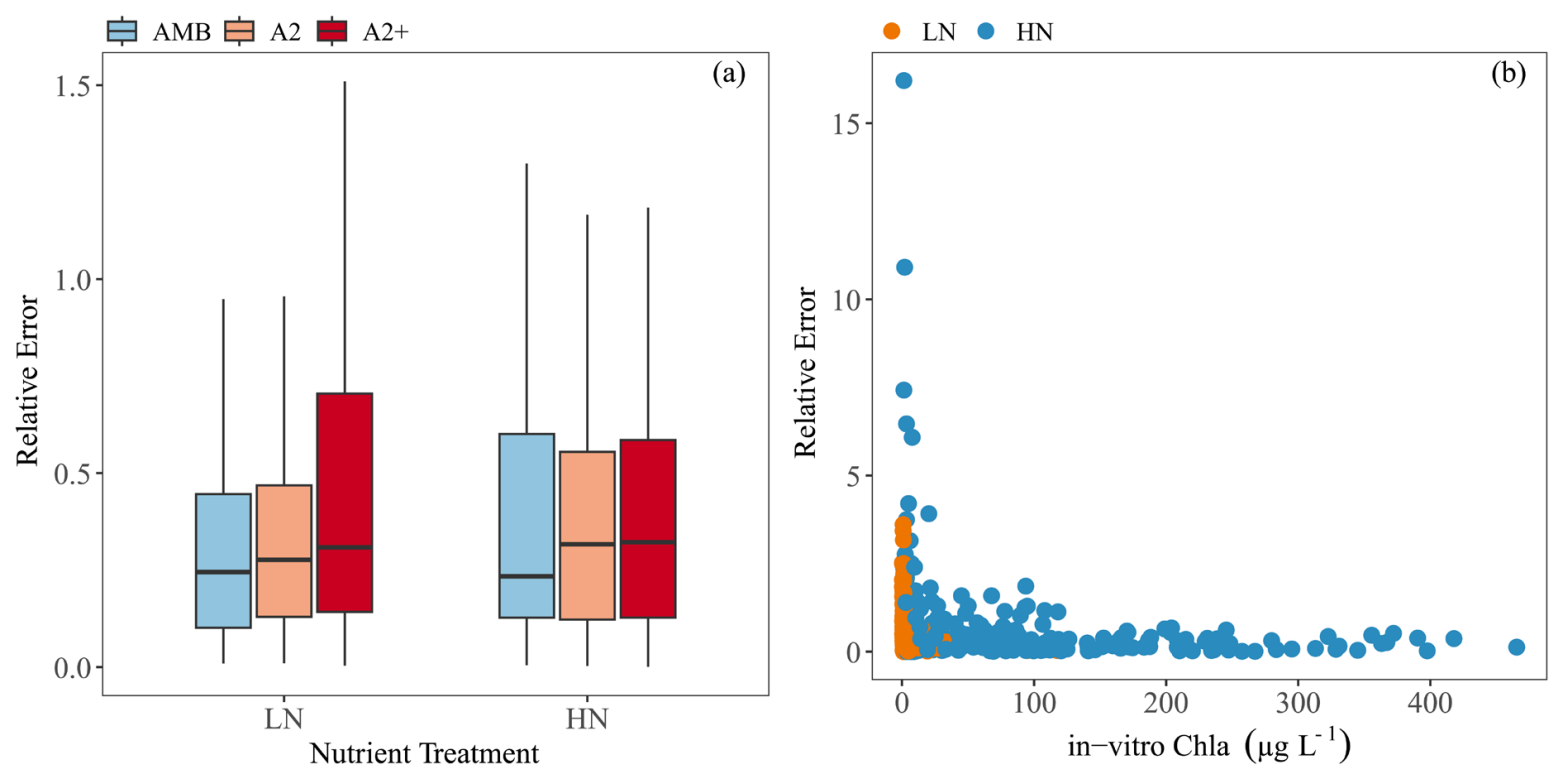
Relative errors (RE) against ( **a**) each nutrient and temperature treatment ( **b**) and in-vitro Chla concentrations. Temperature treatments are Ambient (AMB), A2 (+2-4 °C) and A2+ (+ 4-6 °C). Nutrient treatments are low (LN) and high (HN). Outliers were removed from the boxplot for visual purposes. The boxplots show: minimum and maximum values (range bars), interquartile range (boxes: 25 – 75%), and median value (line within the box). Note the scale differences on the y-axis.

Relative error (RE) was used to compare the performance of the calibration among treatments. Even though RE was highest in the HN tanks, the comparison did not show any significant differences among the treatments; the warmer treatments tended to have a slightly higher RE, though ( Figure 8a). RE was high at low in-vitro Chla particularly in the HN treatments ( Figure 8b).

### Comparing daily Equivalent-Chla with in-vitro Chla

**Figure 9.**
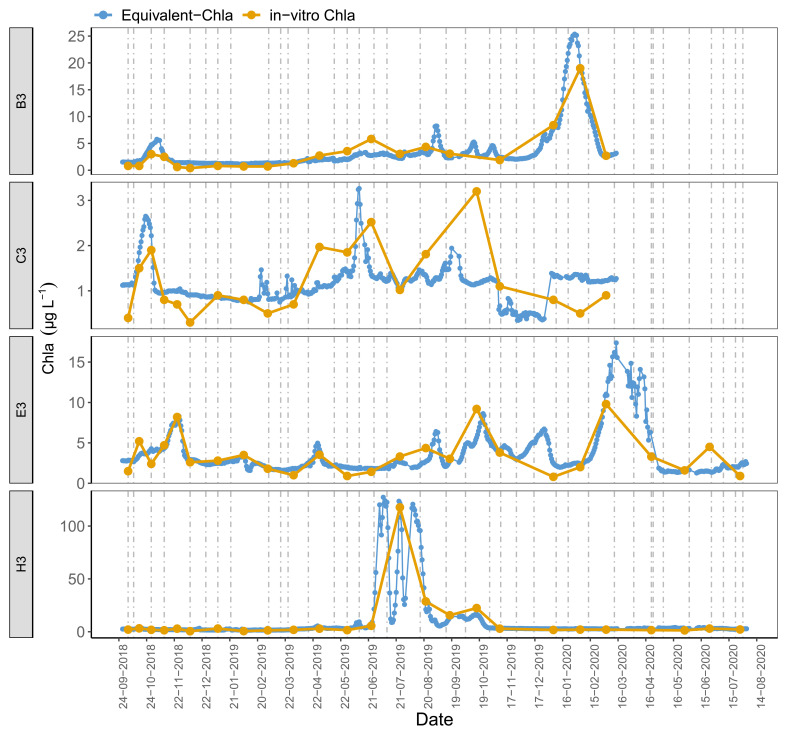
Daily Equivalent-Chla vs in-vitro Chla for low nutrient (LN), A2+ climate scenario treatments. Vertical lines are sensor cleaning days. See AppendixC for figures of the remaining tanks.

**Figure 10.**
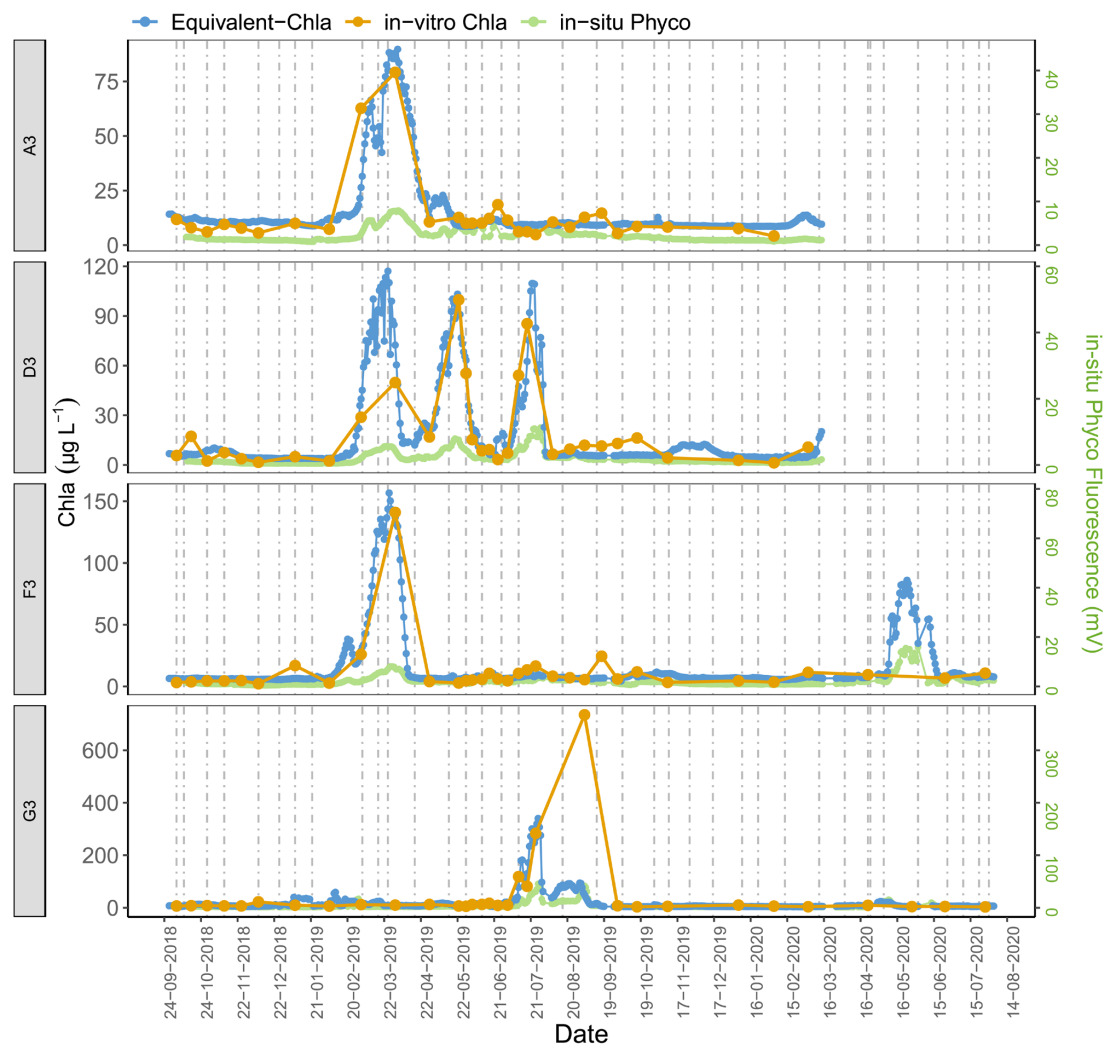
Daily Equivalent-Chla vs in-vitro measurements for high nutrient (HN), A2+ climate scenario treatments. Vertical lines are sensor cleaning days. See AppendixC for figures of the remaining tanks.

The daily Chla concentrations for each tank were predicted for the whole study period (October 2018–August 2020) by using the calibration equations developed in this study between in-vitro Chla and in-situ fluorescence sensor measurements ( Figure 9 and Figure 10, AppendixC). The results illustrate that major events could go unnoticed by the in-vitro Chla measurements. For instance, in tank-H3 (having a high coefficient of determination (r ^2^ = 0.99) between the two measurements), only one of the peaks observed with the in-situ sensors between June and August 2019 was recorded with regular in-vitro sampling ( Figure 9). The tanks with low-moderate explained variance (r ^2^: 0.2 – 0.5) provided similar results, e.g. the small peak at the end of May-2019 in tank C3 was missed by in-vitro sampling.

Observations from high nutrient (HN) tanks were similar ( Figure 10, AppendixC); here Chla peaks were also missed by the in-vitro sampling. Moreover, even if the Chla peaks were caught with the regular in-vitro sampling method, e.g. tank-F3, the true duration of these high-Chla periods was not well captured.

Our results further emphasised the significance of employing multiple regression by including Phyco in-situ fluorescence measurements as a predictor variable in the calibration. For example, around October 2018 in tank-D1, the period with high in-vitro Chla was missed by the in-situ Chla fluorescence sensors but caught by the in-situ Phyco sensor (AppendixD page 1), demonstrating that the high in-vitro Chla reflected an increase in cyanobacteria concentrations. Additionally, as expected, Phyco in-situ fluorescence sensors were also useful in monitoring the changes of relative cyanobacteria concentrations, notwithstanding the lack of calibration. These measurements provide information on the timing of main cyanobacteria changes, while also emphasising the possible impact of in-situ Chla fluorescence on in-situ Phyco values. This can be seen e.g. in tank-D3 where the three in-situ Chla peaks likely caused an increase in in-situ Phyco fluorescence (AppendixD page5).

### Comparing annual and summer means: in-vitro vs Equivalent-Chla

Daily in-vitro Chla concentrations of year-2019 were linearly interpolated, and the obtained values were used to calculate mean annual and summer concentrations for each tank (from here onwards interpolated-Chla _in-vitro_). Similarly, annual and summer means of the Equivalent-Chla concentrations were also calculated. Subsequently, Student’s t-test was used to compare the means between the two methods for each tank.

**Figure 11.**
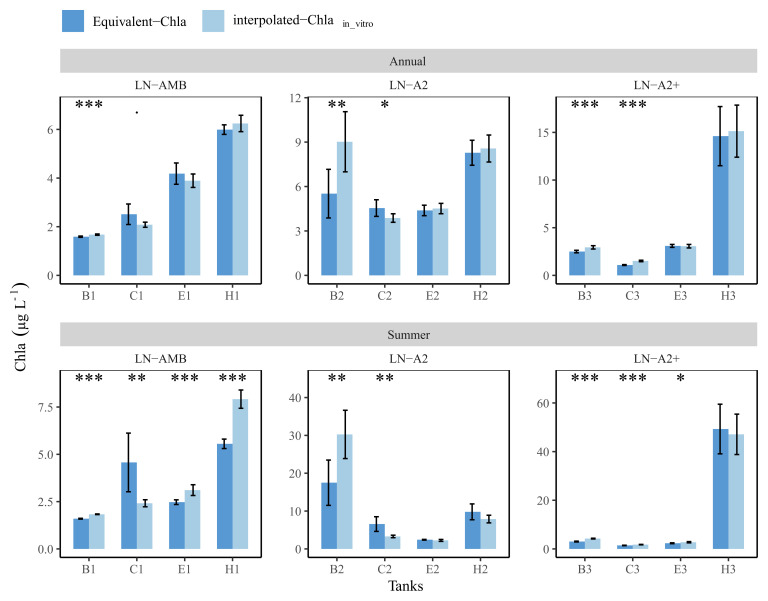
Annual and summer means of Chla concentrations in low nutrient (LN) tanks, calculated based on interpolated-Chla _in-vitro_ and Equivalent-Chla values. Temperature treatments are Ambient (AMB), A2 (+2-4 °C) and A2+ (+ 4-6 °C). Error bars show 95% confidence intervals. The significance ( *p*-value from t-test) is shown as stars where *: *p* < 0.05, **: *p* < 0.01 and ***: *p* < 0.001.

The comparison between the annual mean values with 95% confidence interval (CI) of the two methods revealed no significant differences for many of the low nutrient (LN) tanks. For instance, tank-E1 had annual means (±CI) of 4.2 (±0.4) and 3.9 (±0.3) for Equivalent-Chla and interpolated-Chla _in-vitro_, respectively ( Figure 11). Nevertheless, significant differences were observed especially in the summer means of most of the LN tanks, and the interpolation of in-vitro Chla concentrations either caused over- or under-estimation of the mean values. For example, in tank-B2, both annual and summer means of interpolated-Chla _in-vitro_ were higher than the mean of Equivalent-Chla. Due to linear interpolation, the duration of the interpolated-Chla _in-vitro _peak was wider in comparison to the observed peak width of Equivalent-Chla (AppendixC page4). This was also the case for tank-H1. On the other hand, mean Chla was under-estimated with interpolated-Chla _in-vitro _in tank-C1 because the regular sampling completely missed the Chla peak in May-2019 ( Figure 11 and AppendixC page 3).

**Figure 12.**
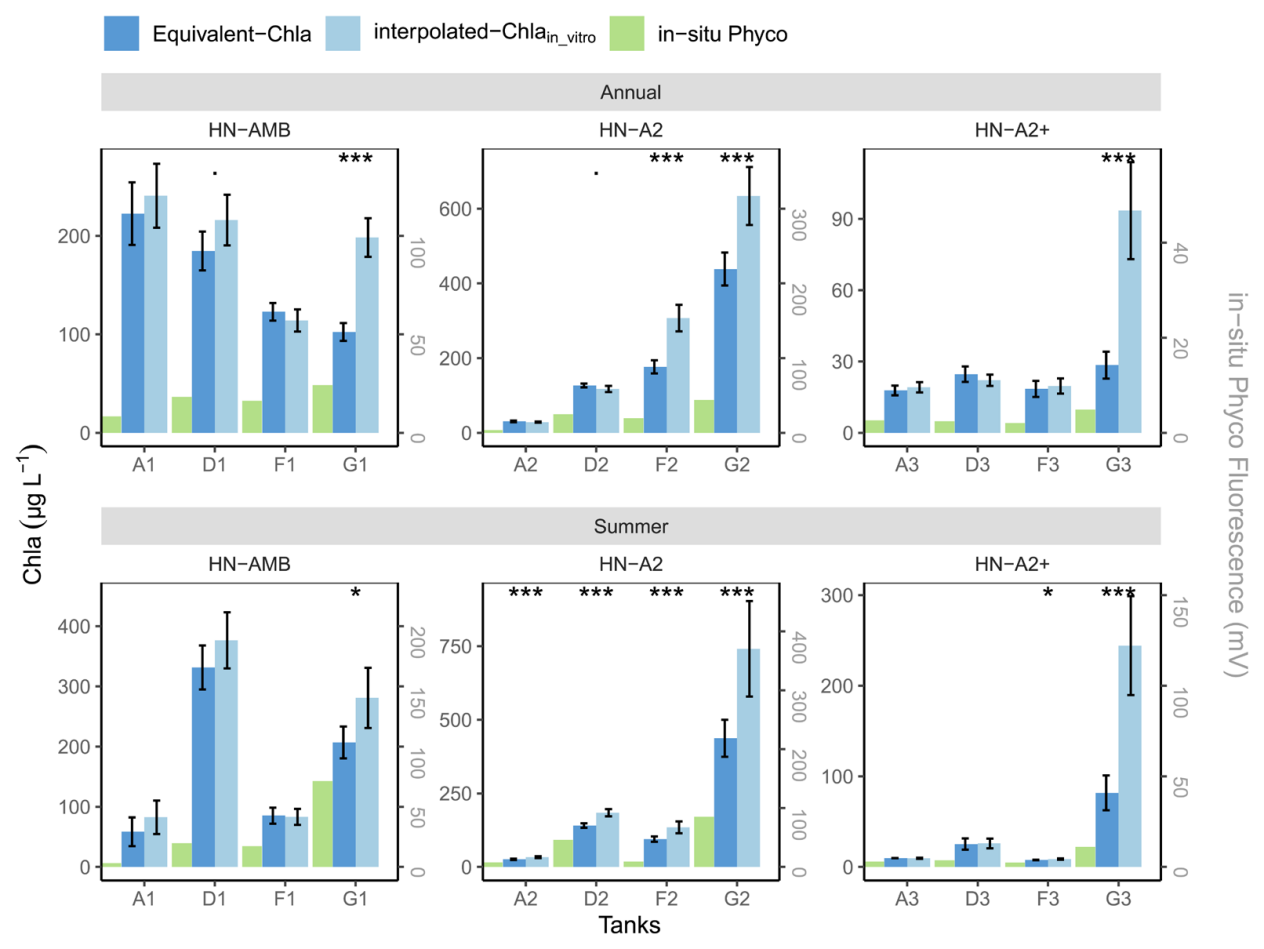
Annual and summer means of Chla concentrations in high nutrient (HN) tanks, calculated based on interpolated-Chla _in-vitro_ and Equivalent-Chla. Temperature treatments are Ambient (AMB), A2 (+2-4 °C) and A2+ (+ 4-6 °C). Error bars show 95% confidence intervals. The significance ( *p*-value from t-test) is shown as stars where *: *p* < 0.05, **: *p* < 0.01 and ***: *p* < 0.001.

The high nutrient (HN) tanks showed a somewhat different pattern ( Figure 12), partly as a consequence of the Chla range limit of the sensors, i.e. concentrations higher than 500 µg Chla L ^-1^ were not measured, but also due to more frequent sampling. Out of eight tanks with high in-vitro Chla concentrations, the mean values from both methods were similar for tanks A1, F1 and partly for D1, while for the other five tanks (G1, D2-F2-G2, G3) mean interpolated-Chla _in-vitro _was significantly higher, both for the summer and annual means ( Figure 12). The tanks (A2, A3-D3-F3) with lower in-vitro Chla concentrations (≤500 µg L ^-1^) mostly had similar annual and summer mean values for both methods, except for A2 and F3 summer. This similarity is likely related to the higher regular in-vitro sampling frequency in the HN compared to the LN tanks, enabling us to capture some of the short-term Chla peaks with regular sampling (e.g. Figure 10 and Figure 12 tank-D3). Despite the better agreement between the two methods for the HN tanks with <500 µg Chla L ^-1^, the positive impact of high frequency (HF) sampling can be observed, like in tank-A2 where regular sampling significantly over-estimated mean summer Chla compared to mean-Equivalent-Chla because it could not catch the sudden, but relatively small, drops in Chla (AppendixC page2).

## Discussion

We tested chlorophyll- *a* fluorescence in-situ sensors over the course of 22 months covering a diverse set of environmental conditions in 24 tanks (mesocosms) simulating fully mixed shallow lakes with contrasting nutrient concentrations and temperatures. The tanks had relatively low and stable concentrations of dissolved organic matter, and turbidity was dominated by phytoplankton. After cleaning of sensor data, the Chla fluorescence in-situ sensors gave reliable concentration estimates; however, the concordance between our Equivalent-Chla concentrations and in-vitro measured values was not perfect since we also observed high relative errors, especially at low Chla concentrations. It is important to recognise that in-situ sensors of Chla fluorescence do not measure Chla concentrations directly.

### Data cleaning: pros and cons

Data-cleaning and interpolation were conducted for all sensors following a comprehensive 11-step procedure (AppendixA). The first steps followed suggestions from the literature, for example removing daytime data, defining a minimum data limit and removing repeated values (e.g. Jaccard *et al.*, 2021), with the aim to remove data recorded during non-photochemical quenching processes as well as negative values and erroneously quenched data. We also used a rate of change (ROC) approach to remove extreme values and to reduce high variation, for example during shade cap-biofouling periods. Jaccard *et al.* (2021) suggested another approach for cleaning Chla in-situ fluorescence data collected from moving platforms in the ocean. They mathematically defined the periods showing a gradual increase of measured values, followed by a sudden drop after sensor cleaning. They were able to identify fouling periods and assigned a possibility scale as higher or lower probability of biofouling ( Jaccard *et al.*, 2021). Although our rate of change approach removed the high variance caused by shade cap biofouling, it was, at least to some extent, not sufficient to identify the in-situ fluorescence decrease caused by the biofouling on the optical parts of the sensors that prevented the transmission of the light beam. Even though biofouling is a well-known issue in water environments, we acknowledge that the challenge we encountered was partly due to the relatively low cleaning frequency of the sensors (mainly 2-week intervals). This issue was mostly prominent in our HN tanks, emphasising that biofouling is a relatively minor issue in oligo- mesotrophic systems.

Following the rate of change approach, data noise and possible remaining anomalies, such as questionable spikes or drops, were filtered out if they were 1.2 times higher or lower than the standard deviations from the rolling 48-hour mean. The latter data cleaning process is widely used for HF time series observations ( Sharma *et al.*, 2010; Teh *et al.*, 2020), but, when used alone, it did not suffice to clean our data when there was a false high variation. This data cleaning procedure resulted in an average data loss of ca. 8% of all night-time 5-minute data collected during the two years.

### Basic inspection of in-situ Chla and Phyco sensors

The first sensor check (linearity check-1) showed, as expected, strongly linear Rhodamine B standard curves. Although the subsequent linearity check-2 indicated more complex conditions in the natural water samples, the sensors were confirmed to be “linear with a proportional increase” within each diluted/undiluted group (Rhodamine dye, low-Chla water sample, high-Chla water sample). A comparison between these groups showed that the increase in in-situ fluorescence values was not proportional. However, optimal fluorescence measurements, e.g. with Rhodamine dye, are not to be expected for water samples due to variation in environmental factors as well as in phytoplankton species composition and size, all being factors that affect the fluorescence signals ( Petersen & Möller, 2017). The comparison of low and high Chla concentration water samples highlighted inconsistencies as the high-Chla in-situ signals were ca. 2.5 times lower than expected compared with the low-Chla in-situ signals when comparing to the respective in-vitro measurements. This was probably due to the relatively high Phyco in-situ fluorescence (ca. 35 mV) in the high-Chla sample, leading to reduced Chla in-situ fluorescence. This is consistent with previous investigations testing the precision of Cyclops-7 fluorescence in-situ sensors, showing a decrease in sensor response with addition of cyanobacterial Chla to a diatom Chla media ( Carroll *et al.*, 2005). Thus, Seppälä *et al.* (2007) found that Chla concentrations could not be estimated by employing only Chla fluorescence measurements during the periods with cyanobacteria blooms in the Baltic Sea. We found similar results in five of twelve HN tanks; using multiple regression, we observed an average 38% increase in coefficient of determination (AppendixB Fig. B5 vs B6). By contrast, Kara *et al.* (2012) did not measure in-situ Phyco fluorescence and observed a very weak relation between in-situ and in-vitro Chla measurements for summer samples (r ^2^ of 0.06, n = 17) from cyanobacteria-dominated Lake Mendota (USA).

### In-situ Chla sensor calibration and evaluating Equivalent-Chla

Overall, we found good correspondence between in-situ Chla fluorescence and in-vitro Chla concentration measurements, especially after employing temperature correction for both in-situ Chla and Phyco fluorescence readings. Linear and multiple regression analyses showed that the coefficient of determination (r ^2^) was >0.5 for 22 of the 24 sensors. Several other studies conducted in lakes ( Kuha *et al.*, 2020), rivers and reservoirs ( Gregor & Maršálek, 2004), fish farms ( Brager *et al.*, 2015) or in the marine environment ( Aiken *et al.*, 2011; Maung-Saw-Htoo-Thaw *et al.*, 2017) have identified similar relationships for in-situ fluorescence and in-vitro measurements, the latter based on HPLC measurements, microscopy or spectrophotometry. It is important to note that the results of these other studies were not obtained using the same brand of sensors and that fluorescence readings can vary, especially among sensors from different manufacturers (e.g. Hodges *et al.*, 2018 for Phyco sensors).

Our results further indicate an impact of phytoplankton composition. Especially in spring, there was a tendency of overestimation of Chla with Equivalent-Chla for both the HN and LN treatments and of underestimation in summer, particularly for HN, possibly reflecting the seasonal variation in phytoplankton communities in these tanks (a shift from a diatom dominated community in spring to cyanobacteria in summer ( Dashkova *et al.*, 2022; Strandberg *et al.*, 2022)). This was supported by the lack of significant differences in relative error between treatments, indicating that the major treatment differences were not the main cause of the error. Lawrenz and Richardson (2011) studied the effect of species composition on Chla fluorescence and showed that a fluorometer calibrated on diatom culture either over- or underestimated the Chla concentration from cultures of dinoflagellates and chlorophytes, respectively. Similarly, Bertone *et al.* (2019) found variations in Phyco in-situ signals between different cyanobacteria cultures of *Sphaerospermopsis* sp., *Aphanocapsa* sp. and *Raphidiopsis raciborskii* compared to total biovolume and to spectrophotometric Phyco measurement.

Shortcomings related to the technical features of the sensors and spectrophotometric methods should also be considered. For example, due to the sensor measurement limit, it was not possible to calibrate the sensors for values higher than 500 µg Chla L ^-1^, resulting in lower Equivalent-Chla concentrations for some of the tanks. The point sampling method also has uncertainties regarding handling, transportation and analysis of the water samples, and together with the lower sensitivity of this method compared to HF fluorescence in-situ sensors ( Lakowicz, 2006; Millie *et al.*, 2010), this may explain the low explained variance in the tanks with low in-vitro Chla concentrations (≤ 5.0 µg L ^-1^). Even though DOC data suggested that the tanks had relatively low and stable concentrations over the two years, we also acknowledge that by not including DOC in the calibration, discrepancies between in-situ fluorescence and in-vitro measurements may have occurred since impact of dissolved organic matter (DOM) on phytoplankton fluorescence has been found in various studies of different sensor brands (e.g. Kuha *et al.*, 2020; Proctor & Roesler, 2010; Twiss, 2011).

### Time series comparison of Equivalent-Chla and interpolated-Chla

High frequency (HF) Equivalent-Chla concentration data allowed us to observe sudden and often large Chla changes (peaks) that were, in part, missed by the point sampling (in-vitro method). These unobserved Chla peaks resulted, in our case, in an underestimation of the mean concentrations of interpolated-Chla _in-vitro_ and were one of the reasons for the significant differences in mean values (annual and summer) between the two methods. Furthermore, HF measurements provided the exact timing of the main changes. Even when the peaks were recorded with the point sampling interpolation of the in-vitro data, the real start and end points of the concentration increase remained undetected; thus, the HF data widened the time period of higher Chla concentrations with implications for the mean values. Overestimation was also found at low Chla concentrations, which might reflect a lower sensitivity of absorbance compared to fluorescence spectroscopy ( Lakowicz, 2006), exhibiting more precise measurement at lower concentrations of in-situ fluorescence methods. Unfortunately, besides the benefits of HF measurements, the upper Chla concentration limit of Cyclops-7F fluorescence in-situ sensors (Turner Designs) prevented correct calculation of the means in many of the HN tanks with >500 µg Chla L ^-1^. Fortunately, such high values are not common in lakes and would thus not be a problem in most cases.

The time series data also highlighted the importance of including in-situ Phyco fluorescence measurements in the calibration as the periods with relatively high in-vitro Chla concentrations and simultaneously low in-situ Chla fluorescence measurements could only be explained by high cyanobacteria concentrations. While multiple regression enhanced the explained variability, it proved inadequate for satisfactory calibration in specific cases, such as tank D2. This limitation may be attributed to the stronger correlation of in-situ Phyco fluorescence with in-vitro Chla and the higher relative change between in-situ Phyco and Chla fluorescence, which exhibited greater variation during the study period compared to other tanks.

## Conclusion

The experimental setup, with two nutrient concentrations and three temperature scenarios (in total six treatments), gave us an advantage in testing the fluorescence in-situ sensors in a diverse set of environments during a ca. two-year period.

In-vitro (spectrophotometric) and in-situ (fluorescence) measurements demonstrated an overall good correlation with occasional exceptions, especially related to sensitivity differences of two methods (fluorescence being more accurate), possibly causing a mismatch regarding low Chla concentrations, and to biofouling on the sensors. Moreover, in-situ Phyco fluorescence (cyanobacteria indicator) was a non-negligible factor that needs to be corrected for when calibrating in-situ Chla fluorescence sensors to compensate for an underestimated cyanobacteria increase by in-situ Chla.

Equivalent-Chla concentrations, calculated from the fluorescence in-situ sensor calibrations, were mostly in agreement with the in-vitro concentrations. Nevertheless, seasonal comparison of Equivalent-Chla and in-vitro concentrations underlined the possible impact by a shift in dominant species during the study period and pointed to overestimated concentrations in spring and underestimation in summer by in-situ sensors, which may be related to a shift from diatom to cyanobacteria communities. To validate phytoplankton seasonality as a source of error in estimating equivalent-Chla concentrations, further studies investigating and comparing the phytoplankton community and its size in the tanks are needed.

High frequency (HF) in-situ fluorescence data, i.e. Equivalent-Chla concentrations, allowed us to observe sudden changes that would otherwise be overlooked in regular sampling and to identify the exact start and end time point of these concentration variations. Furthermore, the investigation and comparison of Equivalent-Chla and interpolated-Chla _in-vitro_ concentrations emphasised the difference between the annual and summer means determined by these two methods. The significant differences in the means were mostly caused by the unobserved Chla peaks or from the misidentified time periods of the sudden changes when using regular low-frequency sampling for in-vitro measurements. The upper Chla concentration limit of the fluorescence sensors was another reason for the disagreement between means as it sets a lower limit to the calibration.

Overall, this study pointed out the importance of frequent Chla recordings despite the known challenges of in-situ fluorescence measurements. We are confident that using a careful approach, fluorescence sensors are a useful tool to monitor sudden and/or short-term changes in aquatic ecosystems. In accordance with previous studies, this study strongly emphasises the need for site-specific calibration as phytoplankton composition and possible interference factors, like DOM, are site-specific. 

## Data Availability

Data available from: Zenodo (RRID:SCR_004129); Lemming Mesocosm (Denmark): in-situ fluorescence chlorophyll- *a* calibration, underlying data, https://doi.org/10.5281/zenodo.10520248 ( Levi *et al.*, 2024a). The datasets listed below are used in; Testing the variability of in-situ fluorescence sensor measurements in Blank (i.e. distilled water) *Data used* : LemCP_BlankFull Testing the linearity (Blank vs Rhodamine B std vs Water samples from the tanks) of in-situ fluorescence sensors *Data used* : LemCP_BlankFull, LemCP_RhodamineFull, LemCP_HighLowChlaSample Testing the impact of DOC concentration on in-situ fluorescence sensor readings *Data used* : LemCP_RhodamineStd, LemCP_RhodamineAddition, LemCP_DOC Linearly interpolating (daily) in-vitro chlorophyll- *a* analysis results from August 2018 until August 2020 *Data used* : LemCP_Spectro_AugSep18, LemCP_Spectro_OctOnw, Taking the night-time median of the cleaned in-situ fluorescence data *Data used* : LemCP_PAR1Med5_QAQCdata, LemCP_DateNewGroup Implementing temperature correction to cleaned in-situ fluorescence data *Data used* : Lem_TempOxyPh (and night-time median in-situ fluorescence data) Calibrating in-situ chlorophyll- *a* sensor data against in-vitro chlorophyll- *a* measurements *Data used* : LemCP_Spectro_OctOnw, (and night-time median in-situ fluorescence data) Marking sensor cleaning dates against biofouling *Data used* : LemCP_SensorCleaning Plotting raw data (i.e. no quality check) *Data used* : LemCP_PAR1Med5_RB3data Metadata file contains detailed information on all the underlying datasets; LemCP_metaData_v1: The metadata comprises 3 sheets, with detailed information on main funding/projects, organization where the study was conducted, datasets used for the current study. Data are available under the terms of the Creative Commons Attribution 4.0 International license (CC-BY 4.0). *Analysis code* Analysis code available from: Zenodo (RRID:SCR_004129), Lemming Mesocosm (Denmark): in-situ fluorescence chlorophyll- *a* calibration analysis code, https://doi.org/10.5281/zenodo.10522849 ( Levi, 2024). Analysis codes used are listed below: LemCP_InSitu_DataClean: These are the rB3 codes that were used to clean the in-situ fluorescence data. LemCP_DataWork_Graphs: This is the main document to prepare the data, calibrate the in-situ fluorescence sensors and to plot all the figures. LemCP_OutliersTest: This is an example (chlorophyll- *a* data collected from tank G2 and A1) of how outliers were defined. Data are available under the terms of the Creative Commons Attribution 4.0 International license (CC-BY 4.0). *Appendix* Appendix tables and figures are available from: Zenodo (RRID:SCR_004129), Lemming Mesocosm (Denmark): in-situ fluorescence chlorophyll- *a* calibration, extended data, https://doi.org/10.5281/zenodo.10523065 ( Levi *et al.*. 2024b). List of appendix included as extended data are as below: AppendA_TableA1: In-situ chlorophyll- *a* and phycocyanin fluorescence data cleaning procedure AppendB_FigB1_Chl_BlankVariation: Blank measurement check; mean and standard deviation of in-situ chlorophyll- *a* and phycocyanin fluorescence sensor (Cyclops-7F, Turner Designs) readings in deionised water (blank sample) for X1 gain and for X10 gain. Note the scale differences on the y-axes. AppendB_FigB2_RhodLinearity: Linearity check-1 for Cyclops-7F in-situ sensors (Turner Designs); Rhodamine B vs in-situ chlorophyll- *a* fluorescence (left pane) and in-situ phycocyanin fluorescence (right pane). Note the scale differences on the y-axes. AppendB_FigB3_LinearReg_LN: Linear regression analysis results for the low nutrient (LN) tanks showing calibration equations and the coefficient of determination (r ^2^) for each tank. Note the scale differences on both axes. (Fluorescence in-situ sensors are Cyclops-7F, Turner Designs). AppendB_FigB4_LinearReg_HN: Linear regression analysis results for 7 of the high nutrient (HN) tanks showing calibration equations and the coefficient of determination (r ^2^) for each tank. Note the scale differences on both axes. (Fluorescence in-situ sensors are Cyclops-7F, Turner Designs). AppendB_FigB5_LinearRegTest_HN-noPhyc: Linear regression analysis results for 5 of high nutrient (HN) tanks showing calibration equations and the coefficient of determination (r ^2^) for each tank. Note the scale differences. (Fluorescence in-situ sensors are Cyclops-7F, Turner Designs). AppendB_FigB6_MultReg_HN: Multiple regression analysis results for 5 of the high nutrient (HN) tanks showing calibration equations and the coefficient of determination (r ^2^) for each tank. The area highlighted in grey indicates the regression plane. Note the scale differences. (Fluorescence in-situ sensors are Cyclops-7F, Turner Designs). AppendC_FigC1_4_Daily_Timeseries: Daily Equivalent-Chla vs in-vitro Chla for the tanks with high (HN) and low (LN) nutrient treatments with ambient (AMB) and IPCC-A2 climate scenario treatments. Vertical lines are sensor cleaning days. AppendD_FigD1_9_RawClean_plots: Plots showing raw data for each tank (i.e. logged every minute and 5 min median taken) against quality-controlled data. Data are available under the terms of the Creative Commons Attribution 4.0 International license (CC-BY 4.0).
