# Supplementary material for: Chlorophyll-a determinations in mesocosms under varying nutrient and temperature treatments: in-situ fluorescence sensors versus in-vitro measurements
Source: Open Res Eur. 2025 Feb 13;4:69. Originally published 2024 Apr 12. [Version 3] doi: 10.12688/openreseurope.17146.3 (PMC11195624; doi:10.12688/openreseurope.17146.3)
Supplement: Peer review history

# Reviewer report: Reviewer response for version 2

**DOI:** 10.21956/openreseurope.20322.r48663

**Published:** 2025-01-11

**Authors:** Kaisa Kraft

**Affiliations:** 1 Marine Research Centre, Helsinki, Finland

The article presents a study comparing continuous fluorometric chlorophyll a measurement with sample-based laboratory analyzed chl a measurements. The measurement have been done in mesocosms with differing nutrient and temperature conditions and therefore differing community compositions. The main aim of the study is to present the advantage of using in situ and continuous fluorometric chl a measurements, also critically addressing the problems related to the method. The study is of high interest to many as in situ chl a fluorescence is a commonly used proxy for algal concentrations. One issue that many face when using the fluorometric data is the proper calibration of the instrument and cleaning of the data, to which the study seeks to answer. The methods were explained thoroughly and discussed well, different aspects were nicely covered.

 I have only two specific comments:

 -In the introduction, the second chapter: Rationale for using in situ fluorometer for chl a detection is that all the other methods (microscopy, HPLC and various forms of flow cytometry) are time consuming, prone to human error, need expertise and require transport to lab for analysis with low spatial and temporal resolution. I am sorry but that is actually not the case. In situ imaging and pulse shape flowcytometry offers a high-resolution in situ alternative with much more detailed information on the community. Of course, they require expertise, but as seen from this study also calibration, maintenance, and post processing of the data from fluorometers also requires expertise. However, fluorometers are much cheaper and easier to use still. But my point was that one cannot say that all listed negative aspects go for all of the listed methods, at least the authors should exclude the automated in situ sensors to make the paragraph be correct and make sense.

 -On page 11, the first chapter of section “Comparing annual and summer means: in-vitro vs equivalent-chla”: This first chapter describes what was done and should belong to the methods section and not the results. Begins with “Daily in-vitro Chla concentrations of year-2019 were linearly interpolated… up till …t-test was used to compare the means between the two methods for each tank.

Is the rationale for developing the new method (or application) clearly explained?

Yes

Is the description of the method technically sound?

Yes

Are the conclusions about the method and its performance adequately supported by the findings presented in the article?

Yes

If any results are presented, are all the source data underlying the results available to ensure full reproducibility?

Yes

Are sufficient details provided to allow replication of the method development and its use by others?

Yes

Reviewer Expertise:

Phytoplankton ecology, imaging flow cytometry, phytoplankton pigments, in situ continuous observations

I confirm that I have read this submission and believe that I have an appropriate level of expertise to confirm that it is of an acceptable scientific standard.

# Response

**Published:** 2025-02-10

**Authors:** Eti Levi

**Affiliations:** Dept. of Ecoscience, Aarhus University, Aarhus, Denmark

*We appreciate the reviewer's time and comments. Below, we have addressed each point separately.* I have only two specific comments:

**-In the introduction, the second chapter: Rationale for using in situ fluorometer for chl a detection is that all the other methods (microscopy, HPLC and various forms of flow cytometry) are time consuming, prone to human error, need expertise and require transport to lab for analysis with low spatial and temporal resolution. I am sorry but that is actually not the case. In situ imaging and pulse shape flowcytometry offers a high-resolution in situ alternative with much more detailed information on the community. Of course, they require expertise, but as seen from this study also calibration, maintenance, and post processing of the data from fluorometers also requires expertise. However, fluorometers are much cheaper and easier to use still. But my point was that one cannot say that all listed negative aspects go for all of the listed methods, at least the authors should exclude the automated in situ sensors to make the paragraph be correct and make sense.**

- *Response* *:  We thank the reviewer for pointing this out. We updated the information by removing the flowcytometry part from the paragraph. The revised sentences now read: “There are several ways to collect information on phytoplankton in waterbodies. Two of the frequently used sampling/analysis methods are microscopy (e.g.* *Hillebrand et al., 1999* *) and pigment analysis with HPLC (* *Roy et al., 2011* *). These methods are especially useful when detailed information on phytoplankton species or groups is required.”*

**-On page 11, the first chapter of section “Comparing annual and summer means: in-vitro vs equivalent-chla”: This first chapter describes what was done and should belong to the methods section and not the results. Begins with “Daily in-vitro Chla concentrations of year-2019 were linearly interpolated… up till …t-test was used to compare the means between the two methods for each tank.**

*Response:  We agree with the reviewer and moved the suggested part to the end of the methods section under* *“* *Annual and summer Chla means: in-vitro vs Equivalent-Chla Comparison”*

# Reviewer report: Reviewer response for version 2

**DOI:** 10.21956/openreseurope.20322.r48263

**Published:** 2024-12-27

**Authors:** Marju Tamm

**Affiliations:** 1 Estonian University of Life Sciences, Tartu, Estonia

This study examines how fluorescence in-situ sensors can be used to gather real-time, high-frequency data for estimating chlorophyll-a (Chla) concentrations. While these sensors are highly useful, converting their fluorescence readings to accurate Chla concentrations is challenging due to factors like biofouling, the composition of phytoplankton, and variations in sensor types.

 To test the reliability of these sensors, researchers compared their readings with traditional spectrophotometric Chla measurements. The study involved 24 freshwater mesocosms, where various combinations of nutrient levels and temperature scenarios were tested.

 Findings were interesting and highly valuable. Most sensors worked well after proper calibration, but there were some exceptions. For example, readings in tanks with high phycocyanin fluorescence or very low Chla concentrations were less reliable. One advantage of using these sensors was their ability to capture sudden shifts in Chla levels, which traditional in-vitro methods often missed due to infrequent sampling. The study also emphasized the importance of cleaning the data to correct errors, such as those caused by environmental interference.

 Overall, the research demonstrates that fluorescence in-situ sensors are valuable tools for monitoring rapid changes in algal biomass. When paired with occasional in-vitro tests, these sensors offer a much more comprehensive picture of what’s happening in freshwater ecosystems.

 The researchers clearly state why the method is needed - there is sufficient discussion about the limitations of the existing methods. The methodological part is clearly explained by the authors and provides sufficient details for replication of the study. All tools, procedures and variables are well-described. The methods used are suitable to address the research objectives.

 Authors have done a very nice job describing all the data collection and analysis methods in sufficient detail. Data cleaning process and statistical analyses are clearly explained and reproducible.

 Although the results of the study do not revolutionise the field, they are still very valuable for the future studies as most of the hydrobiological studies need a good cost-effective determination of algal biomass. The conclusions of this study reflect the data and are clearly supported by it. Potential biases and uncertainties are well-discussed and suggestions for future applications are pointed out clearly.

Is the rationale for developing the new method (or application) clearly explained?

Yes

Is the description of the method technically sound?

Yes

Are the conclusions about the method and its performance adequately supported by the findings presented in the article?

Yes

If any results are presented, are all the source data underlying the results available to ensure full reproducibility?

Yes

Are sufficient details provided to allow replication of the method development and its use by others?

Yes

Reviewer Expertise:

Phytoplankton pigments.

I confirm that I have read this submission and believe that I have an appropriate level of expertise to confirm that it is of an acceptable scientific standard.

# Reviewer report: Reviewer response for version 2

**DOI:** 10.21956/openreseurope.20322.r47759

**Published:** 2024-12-17

**Authors:** Karsten Rinke

**Affiliations:** 1 UFZ-Helmholtz Centre for Environmental Research, Department Lake Research, Brueckstr, Germany

Given the comprehensive revision and overall mature state of the MS I have only two minor comments:

 1.)I wondered about inter-sensor variability in the recorded time series. Do all Cyclops sensors behave the same if proper calibrated or is there notable inter-sensor variability? I am not sure whether the data allow to look for that and it is definitely not an issue that deserves another plot. But a statement would be interesting, if doable with the given data

 2.) Fig4: it is not clear, which x-axis in the plot corresponds to x1 and x2 in the equation

Is the rationale for developing the new method (or application) clearly explained?

Yes

Is the description of the method technically sound?

Yes

Are the conclusions about the method and its performance adequately supported by the findings presented in the article?

Yes

If any results are presented, are all the source data underlying the results available to ensure full reproducibility?

Yes

Are sufficient details provided to allow replication of the method development and its use by others?

Yes

Reviewer Expertise:

aquatic ecology

I confirm that I have read this submission and believe that I have an appropriate level of expertise to confirm that it is of an acceptable scientific standard.

# Response

**Published:** 2025-02-10

**Authors:** Eti Levi

**Affiliations:** Dept. of Ecoscience, Aarhus University, Aarhus, Denmark

We sincerely appreciate the reviewer’s time and feedback. Below, we have provided a response to each point.

 1.) I wondered about inter-sensor variability in the recorded time series. Do all Cyclops sensors behave the same if proper calibrated or is there notable inter-sensor variability? I am not sure whether the data allow to look for that and it is definitely not an issue that deserves another plot. But a statement would be interesting, if doable with the given data

- *Response* *:  We appreciate the reviewer’s question regarding inter-sensor variability. While we did not focus on this aspect in our manuscript, we conducted measurements of a single water sample (10 µg/L Chl-a, determined spectrophotometrically) with 15 of the Chla in-vivo fluorescence sensors. These measurements indicated some variability among the sensors (please see the figure below). The differences may be related to inter-sensor variation, but also to some extent to field conditions as the measurements were not conducted in a controlled environment. *

*Given these factors, we do not feel confident enough to make definitive comments on inter-sensor variability within the manuscript. However, we acknowledge that variability exists and recommend further controlled studies for a more robust answer.*   

 Reference Image: 

https://s3-eu-west-1.amazonaws.com/openreseurope/linked/246441.Reference_Image.pdf 

 2.) Fig4: it is not clear, which x-axis in the plot corresponds to x1 and x2 in the equation

- *Response* *:  We thank the reviewer for pointing this out. We added the information to the caption as; “* *x1 corresponds to in-situ Chla fluorescence and x2 to in-situ Phyco fluorescence.”*

# Reviewer report: Reviewer response for version 2

**DOI:** 10.21956/openreseurope.20322.r46334

**Published:** 2024-12-13

**Authors:** Catherine Schmechtig

**Affiliations:** 1 OSU Ecce Terra, uar3455, Sorbonne Université, Paris, France

The article reports a study that was conducted in mesocosms equipped with fluorometers. The article explains the experimental protocol, the instruments used, all the contaminations they have considered and how they addressed them.  The main conclusion of the article is that using high frequency sensors could bring important information regarding short time events that are not caught by in vitro analysis, assuming that issues like calibration, biofouling, phytoplankton communities, sensor dependant features are considered and corrected (or at least known). The article also highlights the fact that considering cyanobacteria in the study also brought information.

 General remarks :

- I would certainly advocate to add other references to all the different analysis protocols to illustrate that they are shared by a large community.
- I think there should be a paragraph explaining what is the quenching. The term is used several times but without any explanation.

1. “and various forms of flowcytometry” => Flow cytometry in two words
2. In the paragraph, “The importance of open data sources”
  1. I would encourage to add references of open data sources and advocates for example for
    1. Argo http://doi.org/10.17882/42182
    2. Sauzede et al. 2015 https://doi.org/10.5194/essd-7-261-2015
  2. I would also encourage to add the reference Roesler et al., 2017 https://doi.org/10.1002/lom3.10185, to complete the references to address the point that the estimation of Chlorophyll a from fluorescence is strongly sensor dependant
3. In the paragraph, “Sampling for chlorophyll-a: in-vitro determination”,
  1. I would add a short sentence to explain why the sampling frequency changes across seasons
  2. Any other references for the analysis protocol ?
4. In the paragraph, “Chla and Phyco fluorescence sensors: in-situ determination”,
  1. I would add a short sentence to explain Phyco ? previously the short term Chla is explained, but Phyco is not fully explained (is it the short version of phycocyanin ?)
5. In the paragraph, “In-situ sensor data collection and cleaning”,
  1. I assume this sentence is not correct :” PAR values equal to one are used to select night-time in-situ fluorescence sensor data.” Isn’t it PAR values below 1 ? or we set a threshold at 1 ?
6. In the paragraph, “Accuracy of the Equivalent-Chla concentration”
  1. The sentence “though RE was high at low in-vitro Chla particularly in the HN–A2+ treatments (Figure 8b)” is a bit confusing as on the figure 8b, while we can see differences between HN/LN, we cannot see any differences in the plot between AMB, A2 and A2+

Is the rationale for developing the new method (or application) clearly explained?

Yes

Is the description of the method technically sound?

Yes

Are the conclusions about the method and its performance adequately supported by the findings presented in the article?

Yes

If any results are presented, are all the source data underlying the results available to ensure full reproducibility?

Yes

Are sufficient details provided to allow replication of the method development and its use by others?

Yes

Reviewer Expertise:

I am a biogeochemical data manager, so I am quite used to work with fluorometer data, but I am not a biologist so I may miss some expertise regarding phytoplancton communities

I confirm that I have read this submission and believe that I have an appropriate level of expertise to confirm that it is of an acceptable scientific standard.

## References

[1] : Vertical distribution of chlorophyll a concentration and phytoplankton community composition from in situ fluorescence profiles: a first database for the global ocean. *Earth System Science Data* .2015;7(2) : 10.5194/essd-7-261-2015 261-273 10.5194/essd-7-261-2015

[2] : Recommendations for obtaining unbiased chlorophyll estimates from in situ chlorophyll fluorometers: A global analysis of WET Labs ECO sensors. *Limnology and Oceanography: Methods* .2017;15(6) : 10.1002/lom3.10185 572-585 10.1002/lom3.10185

[3] : Author Correction: Floats with bio-optical sensors reveal what processes trigger the North Atlantic bloom. *Nat Commun* .2018;9(1) : 10.1038/s41467-018-04181-0 1834 10.1038/s41467-018-04181-0 29728563 PMC5935696

# Response

**Published:** 2025-02-10

**Authors:** Eti Levi

**Affiliations:** Dept. of Ecoscience, Aarhus University, Aarhus, Denmark

We appreciate the reviewer's time and comments. Below, we have addressed each point separately. General remarks :

- I would certainly advocate to add other references to all the different analysis protocols to illustrate that they are shared by a large community.
  - *Response* *: Please see below for a reference on Chla analysis.*
- I think there should be a paragraph explaining what is the quenching. The term is used several times but without any explanation.
  - *Response* *: We thank the referee for pointing this out. Instead of adding a full paragraph we added some information about quenching.*
    - *In the abstract we included: “*non-photochemical quenching (i.e. light-induced decrease in fluorescence intensity)”
    - *Under the “* *Implementation of the fluorescence in-situ sensors” section, we added: “* *signal-quenching (i.e. reduction in signal intensity due to very high concentrations)”*

 

1. “and various forms of flowcytometry” => Flow cytometry in two words

- *Response* *: Considering another reviewer’s comment this part is removed from the paragraph.*

1. In the paragraph, “The importance of open data sources”
  1. I would encourage to add references of open data sources and advocates for example for
    1. Argo http://doi.org/10.17882/42182
    2. Sauzede et al. 2015 https://doi.org/10.5194/essd-7-261-2015
  2. I would also encourage to add the reference Roesler et al., 2017 https://doi.org/10.1002/lom3.10185, to complete the references to address the point that the estimation of Chlorophyll a from fluorescence is strongly sensor dependant

- *Response* *: We thank the referee for the suggestions. The references are added.*

1. In the paragraph, “Sampling for chlorophyll-a: in-vitro determination”,
  1. I would add a short sentence to explain why the sampling frequency changes across seasons'

- *Response* *: Thank you for pointing this out. The seasonal and treatment-based variation in the sampling frequency was due to a separate, short-term experiment conducted during the same period, which required more detailed monitoring at certain times of the year. Since these details do not directly contribute to the current study's objectives or findings, we opted not to include them in the manuscript.*

-
  1. Any other references for the analysis protocol ?
- *Response* *: We added the reference for Chla extraction by “Jespersen and Christoffersen, 1987)*
  - *Jespersen AM, Christoffersen K: Measurements of chlorophyll a from phytoplankton using ethanol as extraction solvent. Archiv für Hydrobiologie. 1987;109:445–454.*

1. In the paragraph, “Chla and Phyco fluorescence sensors: in-situ determination”,
  1. I would add a short sentence to explain Phyco ? previously the short term Chla is explained, but Phyco is not fully explained (is it the short version of phycocyanin ?)

- *Response* *: We added the short of Phycocyanin in the introduction and also in the suggested paragraph*

1. In the paragraph, “In-situ sensor data collection and cleaning”,
  1. I assume this sentence is not correct :” PAR values equal to one are used to select night-time in-situ fluorescence sensor data.” Isn’t it PAR values below 1 ? or we set a threshold at 1 ?

- *Response* *: We thank the reviewer for the careful check. In our case PAR == 1 is the lowest value we have during night-time. Therefore, PAR values that were equal to 1 were used. *

1. In the paragraph, “Accuracy of the Equivalent-Chla concentration”
  1. The sentence “though RE was high at low in-vitro Chla particularly in the HN–A2+ treatments (Figure 8b)” is a bit confusing as on the figure 8b, while we can see differences between HN/LN, we cannot see any differences in the plot between AMB, A2 and A2+

*Response: We agree with the reviewer and changed the sentence as: “Overall, RE was higher at low in-vitro Chla concentrations (Figure 8b)*”

# Reviewer report: Reviewer response for version 1

**DOI:** 10.21956/openreseurope.18532.r41121

**Published:** 2024-06-18

**Authors:** Michela Rogora

**Affiliations:** 1 National Research Council, Water Research Institute (CNR IRSA), Verbania Pallanza, Italy

The manuscript presents an analysis of fluorescence data from in-situ sensors collected trough an experimental design based on mesocosms with varying nutrient and temperature treatments. Chl-a data gathered from the sensors were tested against in-vitro Chl-a measurements, with the aim to critically assessed sensor data and their applicability in detection and monitoring of algal blooms. The topic covered by the study is of broad interest and results are valuable in assessing the usefulness of in-situ high frequency monitoring in the evaluation of water quality and of potential critical issues. Through the results of their study, the authors present a thorough evaluation of both advantages and limits of high-frequency fluorimetric data, also providing suggestion for a proper management of these data (cleaning and data quality check procedures).

 The manuscript is logically structured, results are extensively and clearly presented and the conclusions sound. I would only suggest a more in-depth discussion on the role of water temperature in the study findings.

 Specific comments:

 In the abstract and introduction there is focus on harmful algal blooms (HABs) as an important threat to freshwater. Phycocyanin, a pigment indicator of cyanobacteria, is also considered in the study; however, even if providing useful information, phycocyanin sensor readings can only suggest the presence (and a biomass estimate) of cyanobacteria. I would stress the importance of in-vitro assessment to confirm the early warning that can be provided by sensor data.

 If I understood correctly, biofouling caused a decrease of the sensor signal, followed by a sudden increase when the optical part of the sensor was cleaned (pag. 9 and 10). We use the same sensors (Cyclops 7, Turner Design, for both Chl and Phycocyanin) for high-frequency monitoring in Lake Maggiore, Italy, and usually get the opposite pattern: fluorescence increases until the upper sensor limit due to biofouling by algae; after the sensor cleaning, the signal immediately goes down. I would suggest to better explain why biofouling caused the signal decrease.

 Temperature scenarios are employed in the treatments together with nutrient levels. However, there are much less results and discussion about temperature effect with respect to high and low nutrient treatments. Is it possible to conclude that temperature has a weaker effect on the performance of in-situ sensors?

 A shift from diatom to cyanobacteria communities is indicated as the possible reason for the overestimation in spring and underestimation in summer of Chl-a concentration by in-situ sensors (pag. 18). This supports the importance of including phycocyanin sensors in high-frequency monitoring programs, as stated in the manuscript. What about phycoerythrin measurements? I think they could be also potentially useful, especially in oligotrophic environments where the contribution of picocyanobacteria to total phytoplankton biomass may be important.

Is the rationale for developing the new method (or application) clearly explained?

Yes

Is the description of the method technically sound?

Yes

Are the conclusions about the method and its performance adequately supported by the findings presented in the article?

Yes

If any results are presented, are all the source data underlying the results available to ensure full reproducibility?

Yes

Are sufficient details provided to allow replication of the method development and its use by others?

Yes

Reviewer Expertise:

Limnology; eutrophication; algal nutrients; chemical loads; lake restoration; high frequency monitoring of lakes; impact of climate change on lakes

I confirm that I have read this submission and believe that I have an appropriate level of expertise to confirm that it is of an acceptable scientific standard.

# Response

**Published:** 2024-10-18

**Authors:** Eti Levi

**Affiliations:** Dept. of Ecoscience, Aarhus University, Aarhus, Denmark

We thank the reviewer for her time and valuable feedback. Below, we have provided responses to each point individually.

1. *In the abstract and introduction there is focus on harmful algal blooms (HABs) as an important threat to freshwater. Phycocyanin, a pigment indicator of cyanobacteria, is also considered in the study; however, even if providing useful information, phycocyanin sensor readings can only suggest the presence (and a biomass estimate) of cyanobacteria. I would stress the importance of in-vitro assessment to confirm the early warning that can be provided by sensor data.*
  - **Response: **We agree with the reviewer and added a sentence at the end of the abstract, which reads; “Nevertheless, corroborating these data with in-vitro Chla and Cyanobacteria assessments would provide additional validation for the early warnings provided by sensor data.”
2. *If I understood correctly, biofouling caused a decrease of the sensor signal, followed by a sudden increase when the optical part of the sensor was cleaned (pag. 9 and 10). We use the same sensors (Cyclops 7, Turner Design, for both Chl and Phycocyanin) for high-frequency monitoring in Lake Maggiore, Italy, and usually get the opposite pattern: fluorescence increases until the upper sensor limit due to biofouling by algae; after the sensor cleaning, the signal immediately goes down. I would suggest to better explain why biofouling caused the signal decrease.*
  - **Response**: That is true that we also observed increased signals with a partial biofouling on the optical part of the sensors. However, we also had fouling fully covering the LED on the optical part which caused a reduction in the received fluorescence signals.
    - This information is added to the paper as: “Biofouling on the optics of the sensors, which fully covered the LED, caused a gradual decrease in in-situ fluorescence values following a sudden increase after sensor cleaning.”
3. *Temperature scenarios are employed in the treatments together with nutrient levels. However, there are much less results and discussion about temperature effect with respect to high and low nutrient treatments. Is it possible to conclude that temperature has a weaker effect on the performance of in-situ sensors?*
  - **Response**: The main reason for the in-depth explanation of high vs. low nutrient tanks was the presence or absence of phycocyanin sensors. Figure 8a indicates no significant difference in relative error between nutrient or temperature treatments, while Figure 8b shows that higher relative error is linked to lower in-vitro Chlorophyll-a concentrations. We have also updated Figure 7 to provide some more detail on temperature treatments. Please see the answer for the other reviewer for more details.
4. *A shift from diatom to cyanobacteria communities is indicated as the possible reason for the overestimation in spring and underestimation in summer of Chl-a concentration by in-situ sensors (pag. 18). This supports the importance of including phycocyanin sensors in high-frequency monitoring programs, as stated in the manuscript. What about phycoerythrin measurements? I think they could be also potentially useful, especially in oligotrophic environments where the contribution of picocyanobacteria to total phytoplankton biomass may be important.*
  - **Response**: Even though in freshwater systems phycoerythrin containing cyanobacteria are typically not dominant, we agree with the reviewer that in environments where picocyanobacteria significantly contribute to the phytoplankton community, measuring phycoerythrin could be important. In such cases, an alternative method may be required for in-vitro Chla analysis, as the method used in the current study does not account for picophytoplankton smaller than 1.2 µm.

# Reviewer report: Reviewer response for version 1

**DOI:** 10.21956/openreseurope.18532.r40259

**Published:** 2024-05-29

**Authors:** Tammi L. Richardson

**Affiliations:** 1 University of South Carolina, Columbia, South Carolina, USA

This is an interesting, well-executed comparison of in-situ vs. in-vitro determinations of chl- *a.* The study leverages an on-going mesocosm experiment with varying nutrient concentrations and temperature. Particular attention was paid to effects of cyanobacteria on chl- *a* estimates, and on data ‘cleaning’ procedures. The multiple regression calibration appears to be successful in explaining additional variance. Overall, I have no major concerns with the data presented or its interpretation.

 Specific Comments

1. Title: The title is long and somewhat overwhelming. Perhaps it could be shortened to something like: “Chlorophyll-a determinations by Cyclops-7F sensors vs. in-vitro measurement under varying nutrient and temperature conditions”.
2. Introduction, second paragraph. I suggest using the word “concentration” or “biomass” to replace the word “biovolume”, as the units are usually expressed in mass not volume.
3. Methods – I suggest adding the duration of the study to the first paragraph of the methods section, i.e., …deployed in October 2018 and recovered in August 2020, and removing it from the paragraph on sampling frequency (as it is a bit confusing there).
4. Methods, 4 ^th^ paragraph: suggest replacing “points” with “depths”.
5. A photograph or sketch of a Cyclops sensor with a shade cap in place would be helpful to understanding the configuration of the Cyclops in each tank.
6. The description of linearity check-2 is a bit confusing. Were chl-a standards used for this calibration? I think it’s the words “used to represent” that are problematic. From the LN tank, were the chl-a concentrations truly 5 and 10 ug/l? (There is similar wording on page 6, second paragraph). Please explain and revise as necessary.
7. Why were Phyco sensors put only in HN tanks?
8. Page 7, please define MM in “MM estimation”.
9. Page 7, DOC effects – Some of the text on DOC could be reduced since there was no substantial DOC interference. This could also be summarized by saying that most DOC in the tank water wasn’t “chromophoric” or “colored” (i.e., CDOM), so not strongly fluorescent.
10. Much of the manuscript focuses on differences among nutrient treatments, but very little on the different temperatures. I know that temperature was used as part of the calibration, but were there any differences among ambient, IPCC, and IPCC+50%? (Apologies if I missed this).
11. In the conclusions, I suggest giving a range of % underestimation to % overestimation for absolute concentrations of chl- *a* by the Cyclops sensors.

Is the rationale for developing the new method (or application) clearly explained?

Yes

Is the description of the method technically sound?

Yes

Are the conclusions about the method and its performance adequately supported by the findings presented in the article?

Yes

If any results are presented, are all the source data underlying the results available to ensure full reproducibility?

Yes

Are sufficient details provided to allow replication of the method development and its use by others?

Partly

Reviewer Expertise:

phytoplankton biology including bulk and spectral fluorometric determinations of phytoplankton biomass and community composition, photosynthesis, primary productivity.

I confirm that I have read this submission and believe that I have an appropriate level of expertise to confirm that it is of an acceptable scientific standard.

# Response

**Published:** 2024-10-18

**Authors:** Eti Levi

**Affiliations:** Dept. of Ecoscience, Aarhus University, Aarhus, Denmark

We appreciate the reviewer's time and comments. Below, we have addressed each point separately.

1. *Title: The title is long and somewhat overwhelming. Perhaps it could be shortened to something like: “Chlorophyll-a determinations by Cyclops-7F sensors vs. in-vitro measurements under varying nutrient and temperature conditions”.*
  - **Response**: We agree with the reviewer and updated the title as suggested with a small change. Now it reads: “Chlorophyll-a determinations in mesocosms under varying nutrient and temperature treatments: in-situ fluorescence sensors versus in-vitro measurements”
2. *Introduction, second paragraph. I suggest using the word “concentration” or “biomass” to replace the word “biovolume”, as the units are usually expressed in mass not volume.*
  - **Response**: We thank the reviewer for spotting this. The word “biovolume” is replaced as “biomass”.
3. *Methods – I suggest adding the duration of the study to the first paragraph of the methods section, i.e., …deployed in October 2018 and recovered in August 2020, and removing it from the paragraph on sampling frequency (as it is a bit confusing there).*
  - **Response**:
    - The first sentence in the methods section is changed as; “The current study was conducted between October 2018 and August 2020 in outdoor mesocosms (from here onwards tanks) at an experimental facility established in 2003 in Lemming, Denmark.”
    - The study period information is removed from “sampling frequency” paragraph.
4. *Methods, 4 ^th^ paragraph: suggest replacing “points” with “depths”.*
  - **Response**: Thank you for the suggestion. Water samples were taken from 3 different “locations” in the tanks, covering the whole water column. In this sentence “points” represent different locations in the tanks. We therefore changed the word “points” as “locations”.
5. *A photograph or sketch of a Cyclops sensor with a shade cap in place would be helpful to understanding the configuration of the Cyclops in each tank.*
  - **Response**: We added the below photograph to the supplementary materials as AppendixE. 17146 Figure 1 URL

1. *The description of linearity check-2 is a bit confusing. Were chl-a standards used for this calibration? I think it’s the words “used to represent” that are problematic. From the LN tank, were the chl-a concentrations truly 5 and 10 ug/l? (There is similar wording on page 6, second paragraph). Please explain and revise as necessary.*
  - **Response:** the word “represent” is removed.
    - The paragraph on page 5 is revised as: “Linearity check-2 was conducted for a subset of the in-situ chlorophyll-a (Chla) sensors. Deionised water served as the blank (0 µg/L). A water sample from an LN tank was used for Chla concentrations of 5 µg/L (1:2 diluted) and 10 µg/L (undiluted). Rhodamine B (Turner Designs) was used for Chla concentrations of 20 µg/L (1:2 diluted) and 40 µg/L (undiluted). Finally, a water sample from an HN tank was used for Chla concentrations of 75 µg/L (1:2 diluted) and 150 µg/L (undiluted).”
    - The part on page 6 is revised as: “These Rhodamine B concentrations are approximate algal Chla equivalents of 10 μg L⁻¹, 20 μg L⁻¹, and 80 μg L⁻¹, respectively”
2. *Why were Phyco sensors put only in HN tanks?*
  - **Response:** Unfortunately, our budget was not enough to have 12 more phycocyanin sensors. Moreover, based on our long-term observations of these mesocosms, we did not expect to have high Cyanobacteria concentrations in low nutrient tanks.
3. *Page 7, please define MM in “MM estimation”.*
  - **Response: **The definition of MM is added to the text as “method of moments (MM) estimation”.
4. *Page 7, DOC effects – Some of the text on DOC could be reduced since there was no substantial DOC interference. This could also be summarized by saying that most DOC in the tank water wasn’t “chromophoric” or “colored” (i.e., CDOM), so not strongly fluorescent.*
  - **Response: **We thank the reviewer for pointing this out. We decided to remove the detailed information about “terrestrial humic-like component” and only focused on the DOC concentrations.
5. *Much of the manuscript focuses on differences among nutrient treatments, but very little on the different temperatures. I know that temperature was used as part of the calibration, but were there any differences among ambient, IPCC, and IPCC+50%?* *(Apologies if I missed this).*
  - **Response**: The main reason for providing a more in-depth explanation of high vs. low nutrient tanks was presence and absence of the phycocyanin sensors in high vs low nutrient tanks, respectively. Overall, Figure 8a shows that the relative error did not differ significantly between the nutrient or temperature treatments. Our results further showed higher relative error to be more related to lower in-vitro Chlorophyll-a concentrations (Figure 8b). Nevertheless, we acknowledge the lack of more in-depth explanation on temperature treatments and have updated Figure7 to address this:

17146 Figure 1 URL 

**Figure 7. Comparison of Equivalent-Chla and in-vitro Chla concentrations, grouped according to season and temperature treatments. **Solid line indicates the 1:1 relation. Nutrient treatments are low (LN) and high (HN). Temperature treatments are ambient (AMB), IPCC-A2 (A2) and IPCC-A2+50% (A2+). The paragraph under “Accuracy of the Equivalent-Chla concentration” section is updated as follows: “The comparison of Equivalent-Chla and in-vitro Chla showed a relatively good 1:1 relation (Figure 7), although some deviation was observed. Especially in spring, there was a tendency to overestimation of Chla by Equivalent-Chla for both the HN and LN treatments, particularly for AMB and A2 (AMB + 2–4 ^o^C) temperature treatments. Moreover, underestimation in the summer, particularly for the HN–A2 treatment (Figure 7) was also observed.

1. *In the conclusions, I suggest giving a range of % underestimation to % overestimation for absolute concentrations of chl-a by the Cyclops sensors.*
  - **Response: **We added information to the second paragraph of the conclusion section. The sentence reads;

“Overall, among the observations with an absolute error larger than the mean absolute error (MAE) of 13, approximately 21% of the data were overestimated, and 25% were underestimated.”
